# Synthesis of triphenylphosphonium vitamin E derivatives as mitochondria-targeted antioxidants

**DOI:** 10.1016/j.tet.2015.09.014

**Published:** 2015-11-04

**Authors:** Victoria J.A. Jameson, Helena M. Cochemé, Angela Logan, Lyall R. Hanton, Robin A.J. Smith, Michael P. Murphy

**Affiliations:** a Department of Chemistry, University of Otago, PO Box 56, Dunedin, 9054, New Zealand; b MRC Clinical Sciences Centre, Imperial College, London, W12 0NN, UK; c MRC Mitochondrial Biology Unit, Hills Road, Cambridge, CB2 0XY, UK

**Keywords:** Mitochondria, Mitochondria-targeted antioxidant, Lipid peroxidation, Vitamin E, MitoE

## Abstract

A series of mitochondria-targeted antioxidants comprising a lipophilic triphenylphosphonium cation attached to the antioxidant chroman moiety of vitamin E by an alkyl linker have been prepared. The synthesis of a series of mitochondria-targeted vitamin E derivatives with a range of alkyl linkers gave compounds of different hydrophobicities. This work will enable the dependence of antioxidant defence on hydrophobicity to be determined in vivo.

## Graphical abstract

**Figure:**
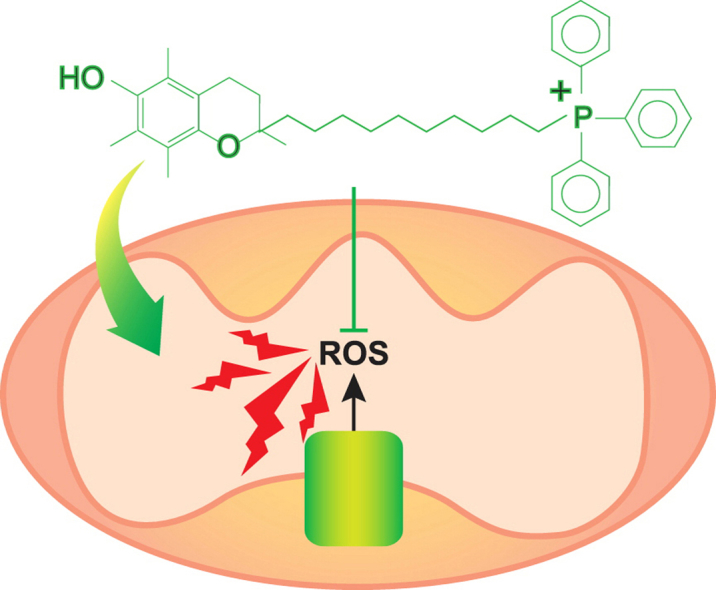

## 1 Introduction

Mitochondria are essential to the functioning of most eukaryotic cells because they provide the energy necessary for cell activities in the form of an elevated adenosine triphosphate/diphosphate ratio by oxidative phosphorylation.^1^ Because free radicals are produced as a side product of this respiration, mitochondrial localised oxidative damage accumulates faster than in the rest of the cell. Mitochondrial dysfunction due to oxidative damage has been implicated in a wide range of conditions from ageing,^2–4^ ischaemia-reperfusion injury,^5–7^ cancer,^8–10^ epilepsy^11^ and to neurodegenerative diseases^12–15^ such as amyotrophic lateral sclerosis^16^ and Alzheimer's^17,18^ and Parkinson's^19^ diseases. Because of the roles mitochondria play in a wide range of pathologies, the engineering of molecules to prevent mitochondrial oxidative damage has therapeutic potential.^20^

Attaching a bioactive moiety to a lipophilic cation, such as triphenylphosphonium (TPP), enables non-mediated, membrane potential driven, accumulation of the active group within the mitochondrial matrix.^21–23^ These molecules have been shown to actively accumulate within mitochondria in tissues following oral delivery.^24^ Numerous examples in the literature, including in vitro,^22,23,25–27^ ex vivo,^28^ in vivo^29^ as well as human trials^30^ have demonstrated that mitochondria-targeted antioxidants are significantly more effective than non-targeted analogues at preventing mitochondrial oxidative damage in a range of pathologies.

To date, mitochondria-targeted antioxidants based on the natural antioxidants Coenzyme Q,^22^ lipoic acid,^31^ and vitamin E^23^ have been synthesised. A series of compounds based on coenzyme Q (*viz.* MitoQ) have been synthesised with 3-,^21^ 5-,^21^ 10-^21,22^ and 15-carbon^21^ alkyl chains linking the TPP and quinone functional groups (MitoQ_3–15_). This set of compounds displayed a wide range of lipophilicities^21^ and there was significant dependence of the protective behaviour in vitro on the linking chain length.^32^ The maximum antioxidant efficacy for the MitoQ_n_ series against mitochondrial oxidative damage was obtained with a 10-carbon alkyl chain (*viz.* MitoQ_10_). This intriguing chain-length dependence was found to be due to increased hydrophobicity which enhanced the extent of uptake into mitochondria by favouring adsorption to the mitochondrial inner membrane; longer linker chain-length which allowed the antioxidant quinol moiety to penetrate deeper into the core of the mitochondrial inner membrane relative to the TPP moiety—which was localized close to the membrane surface; and longer chain length which allowed access of the ubiquinone moiety to the active site of mitochondrial complex II thereby facilitating its rapid reduction to the active ubiquinol antioxidant.^32^ Only one targeted vitamin E molecule (MitoE) has been reported to date and this contains a 2-carbon chain linking the functional, antioxidant chroman moiety of vitamin E to the targeting TPP cation and is thus termed MitoE_2_ (**1**).^23^ As the antioxidant efficacy of the MitoQ_n_ compound depended on alkyl chain length, we have carried out a synthetic study to obtain mitochondria-targeted compounds based on vitamin E with varying alkyl chain lengths, and consequently varying lipophilicities. Previously some of the compounds obtained from this study have been used to create a series of vitamin E succinate derivatives to assay for anticancer activity^33^ and this report also provides full experimental support for the precursors involved in that work.

**Fig. 1:**
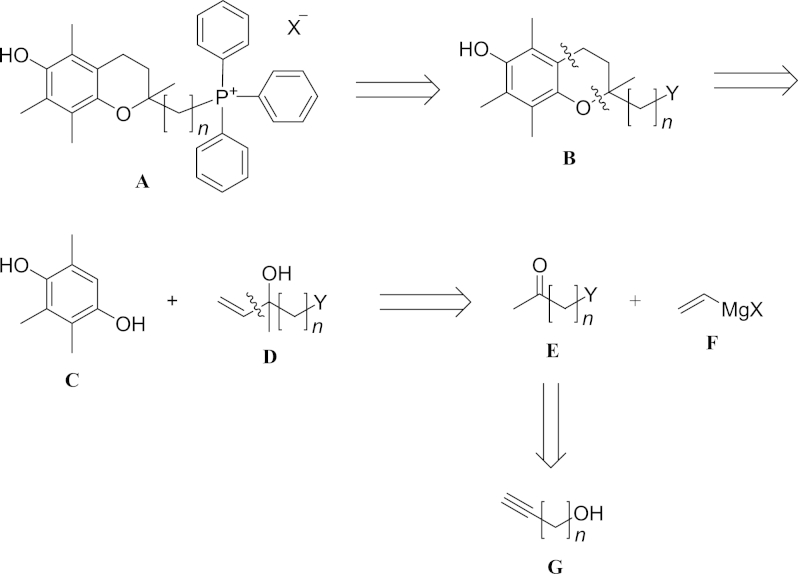
Retrosynthetic analysis of MitoE_n_.

The previous synthesis^23,25^ of MitoE_2_ involved a large number of steps, was not amenable to the creation of analogues, and was not adaptable to scale-up. Retrosynthetic analysis of a generic MitoE_n_ with chain length *n* (**A**) (Fig. 1) shows it can be formed from **B** by displacement of a leaving group Y. The structure **B** is the key intermediate in the synthetic scheme as this establishes the basic carbon framework of the target molecule. The substituted heterocyclic ring in **B** can be derived from 2,3,5-trimethyl-*p*-hydroquinone (**C**) and a tertiary allylic alcohol (**D**) which in turn can be derived from a methyl ketone (**E**) and a vinyl organometallic species (**F**). The appropriate methyl ketone (**E**) can be formed from the corresponding ω-hydroxy alkyne (**G**).

As a variety of ω-hydroxy alkynes are available, especially by utilising acetylene zipper chemistry,^31^ this provides the required flexibility to form any MitoE_n_. Using this general approach we report the synthesis of MitoE_n_ as the mesylate salts with *n*=2, 4, 6 (**1**, **2**, **3**) and also MitoE_10_ (**4**) and MitoE_11_ (**5**) using related chemistry.

## 2 Results and discussion

**Scheme 1:**
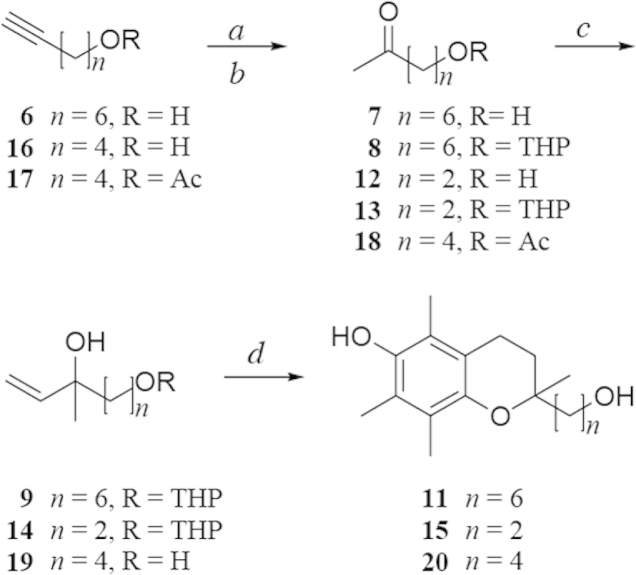
Synthesis of key hydroxychromans for MitoE_2_, MitoE_4_ and MitoE_6_. Reagents and conditions: a HgO, Tf2O, CH3CN (10 min), tetramethylurea (5 min), H2O, CH2Cl2; b DHP, PPTS, CH2Cl2; c vinylMgCl, THF; d HCOOH, 10, reflux.

**Scheme 2:**
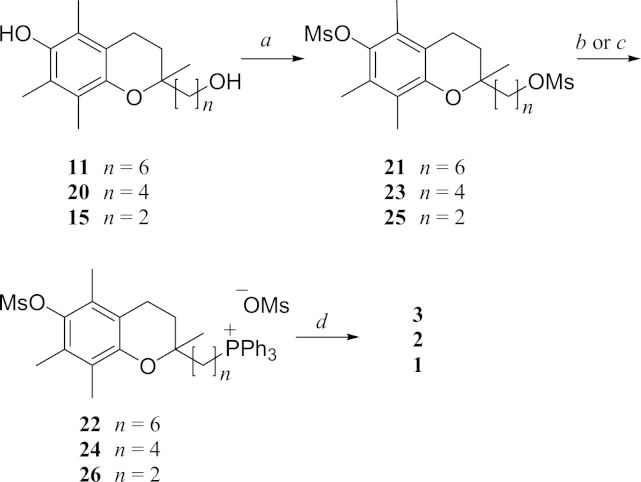
Syntheses of MitoE_2_, MitoE_4_ and MitoE_6_. Reagents and conditions: a MsCl, Et3N, CH2Cl2; b PPh3, 80 C (21 and 23); c PPh3, NaI, 90 C (25); d LDA, THF.

The synthesis of MitoE_6_ (**3**) (Scheme 2), will be described in detail as an exemplar of the methodology. For this the required ω-hydroxy alkyne, 6-octyn-1-ol, (**6**) was treated with Hg(OTf)_2_·(TMU)_2_ in aqueous CH_3_CN^34^ to afford 8-hydroxy-2-octanone (**7**) in 95% yield (Scheme 1). The primary hydroxyl group was then converted into a THP ether (**8**) in 86% yield followed by reaction with vinylmagnesium chloride to readily afford the tertiary allylic alcohol **9**, following chromatography with 0.1% Et_3_N in the elutant, in 96% yield. Reaction of **9** with 2,3,5-trimethyl-*p*-hydroquinone (**10**) in acid^35^—preferably formic acid^36^—afforded diol **11** in 53% yield.

The synthesis of MitoE_2_ and MitoE_4_ followed the same basic route although some specific modifications for producing the key intermediate allylic tertiary alcohol precursors (**14**,**19**) were required reflecting availability of suitable starting materials or undesired intramolecular reactions. Thus the synthesis of the MitoE_2_ hydroxychroman intermediate (**15**, Scheme 1) was completed starting with 4-hydroxy-2-butanone (**12**) and proceeding via the THP ether (**13**) and tertiary allylic alcohol (**14**). The synthesis of MitoE_4_ required the use of 5-hexyn-1-ol (**16**) as the starting material. Reaction of **16** with aqueous Hg(OTf)_2_·(TMU)_2_ resulted in significant formation of the cyclic hemiketal, 2-methyl-tetrahydro-*2H*-pyran-2-ol.^37^ To overcome this undesired cyclization the primary alcohol was converted to an acetate (**17**) before transformation into the acetoxy methyl ketone (**18**).^32^ Treatment of **18** with excess vinylmagnesium chloride simultaneously formed the required tertiary allylic alcohol functionality and hydrolysed the acetate ester affording **19**.

Preliminary studies on the formation of triphenylphosphonium salts from chromanols with an appropriate primary leaving group and triphenylphosphine showed substantial degradation of the heterocyclic system, particularly when the rate of phosphonium salt formation was relatively slow. While the nature of these undesired side reactions was not fully elucidated it was considered useful to protect the electron rich phenol function with an electron withdrawing group during phosphonium salt formation. There are several literature reports of the use of the methanesulfonyl (mesyl) group to protect phenols with subsequent removal using basic reagents.^38^ The mesyl group can therefore serve two functions, as an electrophilic activator of the primary alcohol by forming a mesyloxy group while also providing phenol protection during phosphonium salt formation.

Diol **11** was treated with two equivalents of MsCl in the presence of Et_3_N and afforded the bis-mesylate **21** in 78% yield (Scheme 2). The bis-mesylate was then reacted with PPh_3_ at 90 °C for 48 h to give **22** in 92% yield. Removal of the aryl mesyl ester was trialled with the mesyl derivative of vitamin E and, while reactions using NaBH_4_, Cs_2_CO_3_ or MeONa/MeOH showed no change, reaction with three equivalents of LDA resulted in complete conversion to the phenol vitamin E (Supplementary data). To compensate for the likely ylide formation with the phosphonium substrates, the amount of LDA used in the reaction with **22** was increased to 6 equiv and this protocol afforded MitoE_6_ (**3**) mesylate in 49% yield.

The synthesis of MitoE_4_ (**2**) mesylate was also completed from **20** following the same sequence (*viz.* **20**→**23**→**24**→**2**) (Scheme 2). For the synthesis of MitoE_2_ (**1**) the bis-mesylate (**25**) was readily obtained from **15**, but direct reaction of **25** with PPh_3_ at 80–90 °C produced no phosphonium salt. However addition of 5 equiv of NaI to the melt gave **26**, which was then treated with LDA to afford MitoE_2_ (**1**) mesylate after anion exchange.

During the developmental phase the syntheses of MitoE_10_ (**4**) and MitoE_11_ (**5**) as the mesylate salts were also completed using less efficient earlier variations on the optimised route presented above (Supplementary data). MitoE_10_ (**4**) was synthesised starting from 1-hydroxy-11-dodecyne, obtained by the addition of 1-bromononane to lithiated propargyl THP ether followed by triple bond migration using NaH and ethylenediamine.^39^ In both cases the final step involved reaction of triphenylphosphine with the mesyloxy phenol (S7, S11, Supplementary data) and the adverse effects of extended exposure of the unprotected phenol moiety to these reaction conditions was evident.

**Fig. 2:**
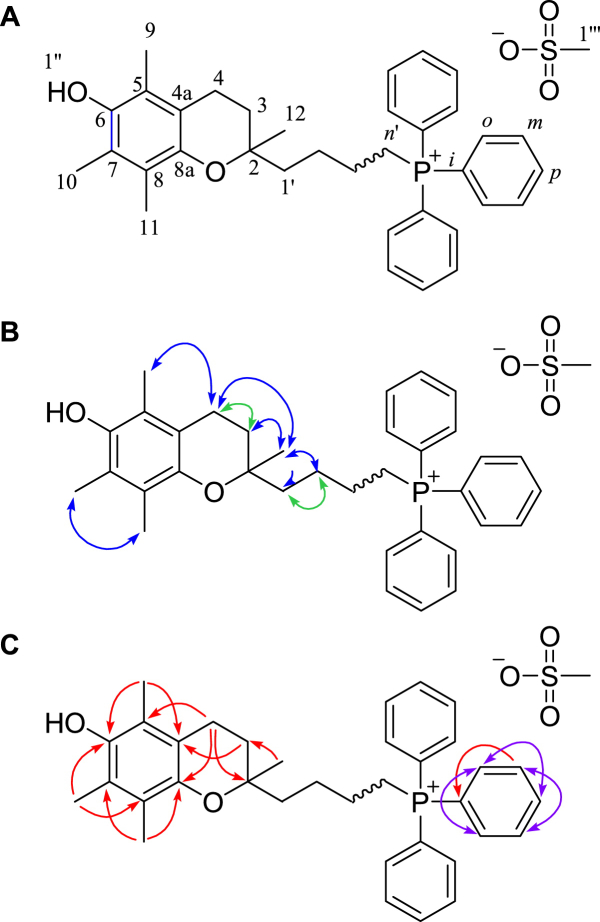
(A) Numbering scheme for assignment of NMR spectra of MitoE_n_. (B) Key H–H correlations:  gCOSY,  NOESY. (C) Key H–C correlations:  HMBC,  mutual HMBC.

The NMR spectra for the mesylates MitoE_2_ (**1**), MitoE_4_ (**2**), MitoE_6_ (**3**), MitoE_10_ (**4**) and MitoE_11_ (**5**) displayed a number of common features which are summarized in Fig. 2 and Table 1. As an example the ^1^H NMR spectrum of MitoE_6_ (**3**) contained a triplet at *δ* 2.55 (*J*=6.8 Hz), consistent with the signal from the methylene group at position 4. NOESY and gCOSY correlations from this resonance to a multiplet at *δ* 1.66–1.80 allowed assignment to the methylene protons at 3. Both of these signals had NOESY correlations to a 3-proton singlet at *δ* 1.17 therefore assigned to the methyl protons at 12. The resonances from the aryl methyl groups were identified from NOESY correlations as from the protons at 10 (*δ* 2.10) and 11 (*δ* 2.00) and a correlation between the signal from the remaining methyl group 9 (*δ* 2.07) and the methylene resonance of 4. The signal from the protons at position 6′ was clearly evident as a multiplet at *δ* 3.22–3.30.

Selected key ^1^H NMR resonances for the mesylates of MitoE_2_, MitoE_4_, MitoE_6_ and MitoE_10_ are summarised in Table 1. The close proximity of the functional groups in MitoE_2_ was evident from the differences compared to the longer chain MitoE compounds.

**Fig. 3:**
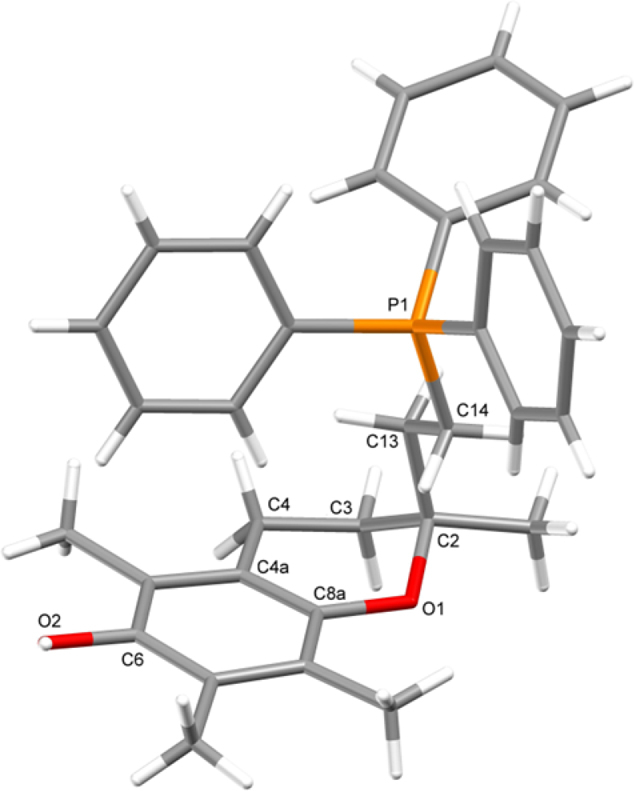
Perspective view of (**1**) showing crystallographic numbering.

Further structural confirmation for MitoE_2_ (**1**) was provided by X-ray diffraction of the crystalline bromide (Fig. 3). In this structure, the six–membered heterocyclic ring of MitoE_2_ adopted an envelope conformation with *θ*=54.5° (lit.^40^ 54.7°) and with C2 deviating from the O1–C3–C4–C4a–C8a plane by 0.699 Å, towards the phosphonium group.

**Fig. 4:**
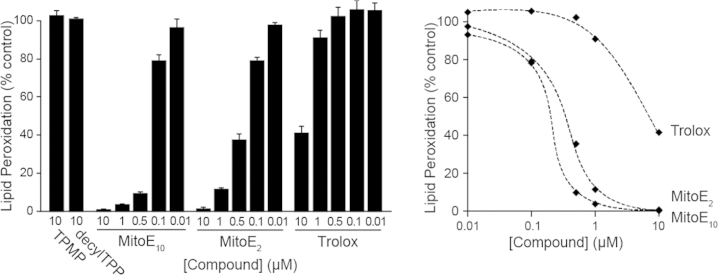
Prevention of lipid peroxidation in rat brain homogenates by MitoE compounds and Trolox.

To determine whether the biochemical properties and antioxidant efficacy of the chromanol moiety of MitoE were affected by changing the length of alkyl chain conjugating it to the TPP cation, the ability of the most (MitoE_10_) and least (MitoE_2_) lipophilic members of the MitoE_n_ series to prevent lipid peroxidation was measured and compared. The ability of the compounds to act as chain breaking antioxidants in the rat brain homogenate system of lipid peroxidation^23^ was assessed. This system was chosen as comparison of the effects of the antioxidants on preventing lipid peroxidation would not be confounded by differential uptake into mitochondria. Rat brain homogenates were allowed to undergo spontaneous lipid peroxidation, which was assessed by the production of thiobarbituric reactive species (TBARS).^22^ The effect of MitoE_2_, and MitoE_10_ on preventing this peroxidation was then assessed (Fig. 4) and showed that both compounds were of comparable efficacy in preventing lipid peroxidation. Therefore it is concluded that conjugation to the TPP cation by differing alkyl chain lengths to the chromanol moiety does not significantly alter their intrinsic antioxidant efficacy. Consequently any changes in antioxidant efficacy seen in mitochondrial or cell studies can be assigned to differences in uptake, adsorption or recycling. The antioxidant efficacy of the MitoE compounds was far greater than that of Trolox, which contains the same antioxidant chromanol moiety as MitoE, connected to a short-chain carboxylic acid rather than a TPP function, making it far more hydrophilic. This suggests that the lipophilic nature of the TPP moiety enhances the interaction of the antioxidant moiety with the phospholipid bilayer.

**Fig. 5:**
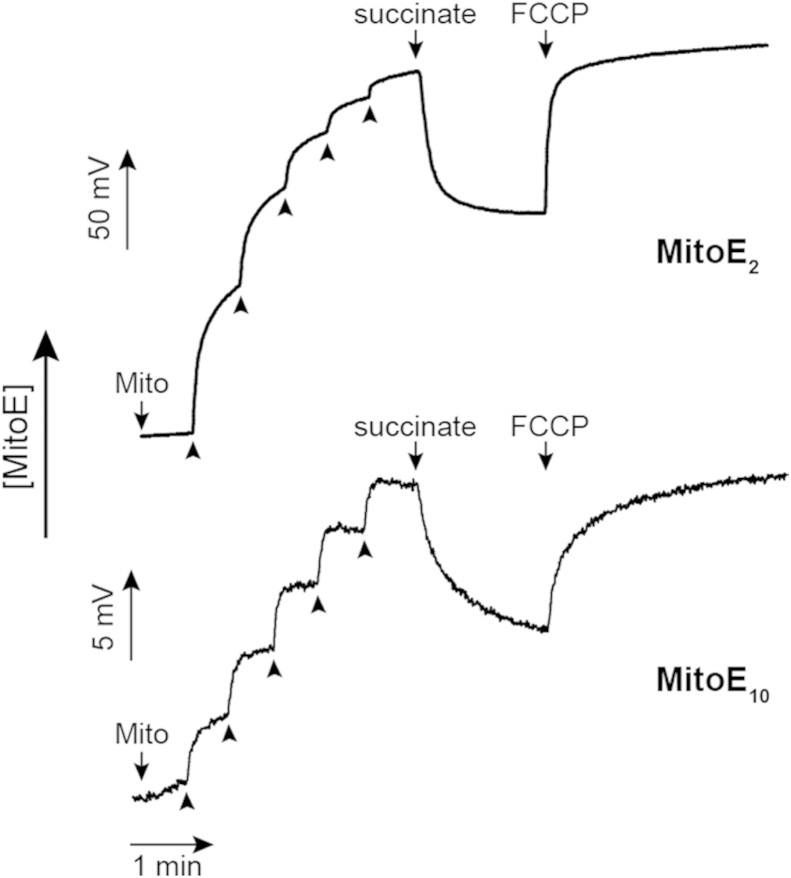
Accumulation of MitoE_2_ and MitoE_10_ by isolated rat liver mitochondria (Mito) energised with the respiratory substrate succinate and their release upon dissipation of the mitochondrial membrane potential with the uncoupler carbonyl cyanide *p*-trifluoromethoxyphenylhydrazone (FCCP).

This aspect was extended by measuring whether MitoE_2_ and MitoE_10_ compounds were accumulated by mitochondria in response to the mitochondrial membrane potential, as is expected for a compound linked to a TPP compound. To measure the uptake of the MitoE compounds an ion-selective electrode was used that responds to the concentration of the TPP cation in solution (Fig. 5). When the MitoE compounds were added to a mitochondrial suspension the ion-selective electrode responded to the increase in concentration of the MitoE compound in the extracellular environment. When the mitochondria were energised with the respiratory substrate succinate a large membrane potential across the mitochondrial inner membrane was established. This led to the extensive accumulation of the compounds within mitochondria, thus lowering the extracellular concentration which is detected by the electrode. Addition of the uncoupler FCCP resulted in the dissipation of the membrane potential and consequent release of the compounds back in to the supernatant and this is evident from the electrode response.

**Fig. 6:**
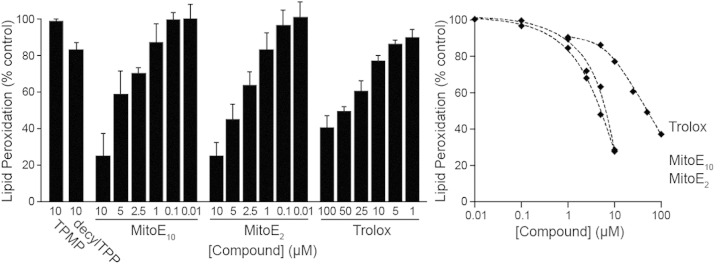
Prevention of lipid peroxidation in rat liver mitochondria by MitoE_2_, MitoE_10_ and Trolox.

Therefore these experiments showed that, as expected, both MitoE_2_ and MitoE_10_ were taken up by energised mitochondria in response to the membrane potential. To see if this accumulation within mitochondria enhanced the ability of the mitochondria-targeted compounds to decrease oxidative damage to isolated mitochondria the activity was assessed and compared with the antioxidant efficacy of Trolox, a chroman containing antioxidant molecule, which is not taken up by mitochondria (Fig. 6).

**Fig. 7:**
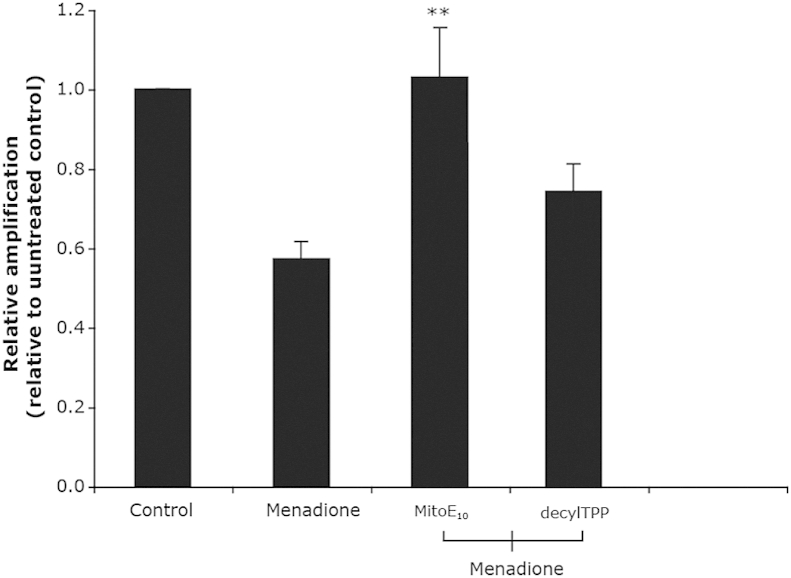
Prevention of oxidative damage to mitochondrial DNA in cells in culture by MitoE_10_. The lower the relative amplification the greater the damage to DNA.

This showed that both MitoE_2_ and MitoE_10_ were comparably protective against mitochondrial lipid peroxidation and that both were more protective against oxidative damage that Trolox. Finally, the ability of the most effective version of MitoE, MitoE_10_ to protect against oxidative damage to mitochondrial DNA caused by the redox cycling molecule menadione was determined (Fig. 7). This also showed that MitoE_10_ was able to protect against this form of mitochondrial oxidative damage more effectively that the control compound decylTPP. MitoE_2_ was not protective in this assay (data not shown) consistent with the greater protection of MitoE_10_ against lipid peroxidation.

## 3 Conclusion

The development of this generalised route to MitoE analogues has allowed a suite of targeted analogues to be prepared. We have assessed the biochemical properties of the most (MitoE_10_) and least (MitoE_2_) lipophilic members of the MitoE compounds and found that they are effective. By analogy with the MitoQ suite of compounds the series of MitoE compounds we have made will also have a range of lipophilicities and can be used to assess the effect of lipophilicity on the biological effects of MitoE. This strategy is both efficient and can be systematically varied to create a range of MitoE compounds. These compounds accumulate in mitochondria and preliminary biological data demonstrate that MitoE analogues show greater efficacy in preventing lipid peroxidation, mitochondrial oxidative damage and damage to mitochondrial DNA than non-targeted compounds. Further work needs to be conducted to fully understand the trend of biological behaviour of the MitoE series and determine the activity of the intermediate MitoE compounds and enable the dependence of the antioxidant efficacy of MitoE on chain length to be assessed in vivo.

## 4 Experimental section

### 4.1 General procedures

Thin Layer Chromatography (TLC) was performed with silica gel (Merck) 60F 254 coated on aluminium roll and were developed in solvent mixtures as indicated. Plates were visualised first with UV light (254 nm) then stained with vanillin or phosphomolybdic acid and heated. Column chromatography was performed using Merck 60 Silica (200–400 mesh, 40–63 μm) as the adsorbent. Columns were pre-equilibrated with the starting solvent before use and 50 g of adsorbent per g of crude product was used. Anion Exchange Chromatography was performed using Amberlite^®^ IRA-400(Cl) ion exchange resin. The column was pre-equilibrated with 10% aqueous MsOH before use. Material was loaded in MeOH and the column was eluted with 1:1 MeOH:H_2_O.

Nuclear Magnetic Resonance (NMR) Spectroscopy ^1^H and ^13^C NMR spectra were acquired on a Varian INOVA-300 spectrometer at 7.05 T and 298 K operating at 299.90 and 75.42 MHz, respectively, or on a Varian INOVA-500 spectrometer at 11.74T and 298 K, operating at 499.74 MHz and 125.67 MHz, respectively as indicated. ^31^P NMR spectra were acquired on a Varian INOVA-300 spectrometer at 7.05 T and 298 K operating at 121.40 MHz. Spectra were acquired in CDCl_3_, CD_2_Cl_2_ and CD_3_OD as indicated. The solvent peak was used as an internal reference for ^1^H and ^13^C NMR spectra. In CDCl_3_ the ^1^H and ^13^C NMR spectra were referenced to 7.26 ppm and 77.08 ppm, respectively; in CD_2_Cl_2_^1^H and ^13^C NMR spectra were referenced to 5.31 ppm and 53.8 ppm, respectively and CD_3_OD to 3.31 ppm and 49.0 ppm, respectively. Phosphoric acid (85%) was used as an external reference at 0 ppm for ^31^P NMR spectra. Spectra were processed using standard Varian software. For each ^1^H NMR signal, chemical shifts (*δ*), relative integral, multiplicity, coupling constant (*J*), and assignment information are given unless otherwise stated. ^13^C resonances were evident as singlets from a single carbon unless otherwise stated. The following standard abbreviations are used: s=singlet, d=doublet, t=triplet, m=multiplet, dd=doublet of doublets, td=triplet of doublets, dt=doublet of triplets. Standard 2D experiments (gCOSY, NOESY, HSQC and HMBC) were employed for assignment of proton and carbon resonances.

Low resolution atmospheric pressure chemical ionisation (APCI) mass spectroscopy (MS) and low resolution electrospray ionisation (ESI) MS were performed using a Shimadzu LCMS WP8000α spectrometer operated in positive or negative ion mode as indicated. High resolution mass spectra (HRMS) were recorded on a Bruker microTOF-Q spectrometer operated in positive or negative ion mode as indicated. Data are presented as *m/z* values for the parent molecular ion. Combustion microanalyses were performed by M. Dick or R. McAllister (Campbell Microanalytical Laboratory, Department of Chemistry, University of Otago).

X-ray diffraction data were collected on a Bruker APEX II CCD diffractometer, with graphite monochromated Mo-Kα (*λ*=0.71073 Å) radiation. Intensities were corrected for Lorentz polarisation effects and a multiscan absorption correction was applied.^41^ The structure was solved by direct methods (SIR–97^41^) and refined on *F*^2^ using all data by full-matrix least-squares procedures (SHELXL 97^42^).

### 4.2 Syntheses

#### 4.2.1 General synthetic procedures

Argon was used for reactions requiring an inert atmosphere. Standard vacuum line Schleck techniques were employed; glassware was flame-dried before use and cannula were used for transferring liquids between reaction vessels. Removal of solvents was achieved by either rotary evaporation at temperatures of up to 50 °C, or by evaporation under a stream of argon. Some syntheses were carried out a 15 mL Kimax brand round bottomed 10 mm id borosilicate test-tubes fitted with a screw-top that enables a convenient sealed system under argon to be established.

Diisopropylamine was distilled from NaOH before use. Trifluoromethanesulfonic anhydride (Tf_2_O) was distilled from P_2_O_5_ immediately before use. 2,3,5-Trimethyl-*p*-hydroquinone (**10**) was stirred as a suspension in hexane (10 equiv w/v) was stirred for 30 min. The solvent was removed by filtration and the pale yellow precipitate was dried at <0.5 mmHg for 2 h. Pyridine was refluxed over KOH and distilled onto 4 Å molecular sieves before use. All other reagents were used without purification.

Absolute alcohol was dried over 4 Å molecular sieves. Acetone was refluxed then distilled onto 3 Å molecular sieves. Acetonitrile (CH_3_CN) was refluxed over CaH_2_ and distilled directly before use. Dichloromethane (CH_2_Cl_2_) was refluxed over P_2_O_5_ and distilled onto 3 Å molecular sieves. Diethyl ether (Et_2_O) was distilled before use and stored over sodium wire. Tetrahydrofuran (THF) was refluxed over KOH then distilled onto Na wire. The pre-dried solvent was then freshly distilled under an argon atmosphere over a benzophenone-K-Na amalgam (4:1) prior to use. All other solvents were used without purification.

#### 4.2.2 9-(Tetrahydro-2*H*-pyran-2-yloxy)-3-hydroxy-3-methyl-non-1-ene (**9**)

Vinylmagnesium chloride (1.4 M in THF, 6.3 mL, 8.82 mmol) was added to a solution of **8** (Supplementary data) (1.006 g, 4.41 mmol) in anhydrous THF (60 mL) stirring at −78 °C. This was stirred for 2 h and then allowed to warm to room temperature over 30 min. To the reaction mixture was added dropwise saturated aqueous NH_4_Cl (50 mL) and then this was extracted with Et_2_O (3×50 mL). The combined organic phase was washed with saturated aqueous NaCl (50 mL), dried over anhydrous MgSO_4_, filtered and concentrated to give **9** as a pale yellow oil (1.086 g, 4.24 mmol, 96%) which was used without further purification. Analysis calcd for C_15_H_28_O_3_: C 70.3, H 11.0, found: C 70.3, H 10.9; TLC: R_f_ 0.37 (1:9 Et_2_O:CH_2_Cl_2_); HRMS (+ve ESI) *m/z* calcd for [M+Na]^+^:279.1931, found: 279.1933; ^1^H NMR (500 MHz, CD_2_Cl_2_): *δ* (ppm) 1.22 (3H, s, **H15**), 1.26–1.38 (6H, m, **H5**–**H7**), 1.44–1.60 (8H, m, **H4**, **H8**, **H11a**, **H12a**, **H13**), 1.62–1.88 (1H, m, **H11b**), 1.74–1.82 (1H, m, **H13b**), 3.33 (1H, td, *J*_HCH_=9.6, *J*_HCCH_=6.7 Hz, **H9a**), 3.42–3.46 (1H, m, **H14a**), 3.66 (1H, td, *J*_HCH_=9.6, *J*_HCCH_=6.7 Hz, **H9b**), 3.81 (1H, ddd, *J*=11.3, 8.3, 3.0 Hz, **H14b**), 4.52 (1H, t, *J*=3.7 Hz, **H10**), 5.00 (ABX system, 1H, dd, *J*_AX_=10.7 Hz, *J*_AB_=1.4 Hz, **H1a**), 5.16 (ABX system, 1H, dd, *J*_BX_=17.4 Hz, *J*_AB_=1.4 Hz, **H1b**), 5.90 (ABX system, 1H, dd, *J*_BX_=17.4 Hz, *J*_AX_=10.7 Hz, **H2**); ^13^C NMR (125 MHz, CD_2_Cl_2_): *δ* (ppm) 20.1 (**C12**), 24.2 (**C5**), 26.0 (**C13**), 26.6 (**C7**), 27.9, 27.9 (1C, 2× s, **C15**), 30.1 (**C8**), 30.3 (**C6**), 31.2 (**C11**), 42.8 (**C4**), 62.4 (**C14**), 67.8 (**C9**), 73.4 (**C3**), 99.1 (**C10**), 111.3 (**C1**), 146.0 (**C2**).

#### 4.2.3 2-(6-Hydroxyhexyl)-2,5,7,8-tetramethyl-chromen-6-ol (**11**)

A solution of **9** (0.959 g, 3.74 mmol) and freshly prepared 2,3,5-trimethyl-*p*-hydroquinone (**10**, 0.460 g, 3.02 mmol) in formic acid (90 mL) were heated to reflux and refluxed for 3 h under an atmosphere of argon. The reaction was poured into crushed ice (∼180 mL) and this was extracted with Et_2_O (3×100 mL) under argon. The combined organic phase was washed with H_2_O (3×100 mL) under argon, dried over anhydrous MgSO_4_ and concentrated. The oily brown residue was dissolved in MeOH (90 mL), conc. HCl (0.090 mL) was added and the reaction refluxed for a further 30 min under argon. The reaction was diluted with ice cold H_2_O (200 mL) and this was extracted with Et_2_O (3×100 mL) under argon. The combined organic phase was washed under argon with H_2_O (3×400 mL), saturated aqueous Na_2_CO_3_ (3×100 mL) and H_2_O again (3×100 mL), dried over anhydrous MgSO_4_, filtered and concentrated to give a brown oil. The crude product was purified by column chromatography on silica gel and elution with 1:9 Et_2_O:CH_2_Cl_2_ followed by crystallisation from CH_2_Cl_2_ and afforded **11** as a pale yellow solid (0.490 g, 1.60 mmol, 53%). TLC: *R*_*f*_ 0.29 (1:9 Et_2_O:CH_2_Cl_2_); HRMS (+ve ESI) *m/z* calcd for [M+Na]^+^: 329.2087, found: 329.2068; HRMS (−ve ESI) *m/z* calcd for [M−H]^+^: 305.2122, found: 305.2135; ^1^H NMR (500 MHz, CDCl_3_): *δ* (ppm) 1.22 (3H, s, **H12**), 1.28–1.46 (6H, m, **H2′**–**H4′**), 1.28–1.46 (4H, m, **H2′**, **H5′**), 2.105 (3H, s, **H11**), 2.105 (3H, s, **H9**), 2.16 (3H, s, **H10**), 2.60 (2H, t, *J*=7.0 Hz, **H4**), 3.62 (2H, t, *J*=6.3 Hz, **H6′**), 4.2 (1H s (br), **H7′**); ^13^C NMR (125 MHz, CDCl_3_): *δ* (ppm) 11.3 (**C9**), 11.8 (**C11**), 12.3 (**C10**), 20.8 (**C4**), 23.6 (**C2′**), 23.8 (**C12**), 25.8 (**C4′**), 30.0 (**C3′**), 31.6 (**C3**), 32.8 (**C5′**), 39.5 (**C1′**), 63.1 (**C6′**), 74.5 (**C2**), 117.4 (**C4a**), 118.6 (**C5**), 121.1, 122.7 (2C, 2× s, **C7**, **C8**), 144.6 (**C6**), 145.6 (**C8a**).

#### 4.2.4 5-(Tetrahydro-2*H*-pyran-2-yloxy)-3-hydroxy-3-methyl-pent-1-ene (**14**)

Vinylmagnesium chloride (1.40 M in THF, 9.5 mL, 13.3 mmol) was added to a solution of **13** (Supplementary data) (1.006 g, 5.84 mmol) in anhydrous THF (50 mL) stirring at −78 °C. This was stirred for 2 h and then allowed to warm to room temperature over 30 min. Saturated aqueous NH_4_Cl (50 mL) was added dropwise and this was extracted with Et_2_O (3×50 mL). The combined organic phase was washed with saturated aqueous NaCl (50 mL), dried over anhydrous MgSO_4_, filtered and concentrated to give a pale yellow oil. The crude product was purified by column chromatography on silica gel and elution with 1:3 Et_2_O:CH_2_Cl_2_ containing 0.1% Et_3_N afforded pure **14** as a colourless liquid (1.091 g, 5.45 mmol, 93%). Analysis calcd for C_11_H_20_O_3_: C 66.0, H 10.1, found: C 65.9, H 10.2; TLC: *R*_*f*_ 0.71 (1:1 Et_2_O:CH_2_Cl_2_); HRMS (+ve ESI) *m/z* calcd for [M+Na]^+^: 223.1305, found: 223.1305; ^1^H NMR (500 MHz, CDCl_3_): *δ* (ppm) 1.28, 1.29 (3H, 2× s, **H11**), 1.48–1.63 (4H, m, **H7a**, **H8a**, **H9**), 1.66–1.82 (3H, m, **H4a**, **H7b**, **H8b**), 1.92–1.99 (1H, m, **H4a**), 3.47, 3.49 (1H, 2× s, **H11**), 3.48–3.57 (2H, m, **H5a**, **H10a**), 3.79–3.86 (1H, m, **H10b**), 3.92–3.98 (1H, m, **H5b**), 4.57, 4.60 (1H, 2× t, *J*=7 Hz, **H6**), 5.06–5.11, 5.27–5.93 (2H, 2× m, **H1**), 5.86–5.93 (1H, m, **H2**); ^13^C NMR (125 MHz, CDCl_3_): *δ* (ppm) 19.3, 19.2 (1C, 2× s, **C8**), 25.3(5), 25.3(7) (1C, 2× s, **C9**), 28.6, 28.7 (1C, 2× s, **C11**), 30.4, 30.6 (1C, 2× s, **C7**), 40.2(5), 40.3(2) (1C, 2× s, **C4**), 61.9, 62.1 (1C, 2× s, **C10**), 64.9, 65.2 (1C, 2× s, **C5**), 73.4 (**C3**), 98.7, 98.9 (1C, 2× s, **C6**), 112.1, 112.3 (1C, 2× s, **C1**), 144.6 (**C2**).

#### 4.2.5 3,7-Dihydroxy-3-methyl-hept-1-ene (**19**)

Vinylmagnesium chloride (1.4 M in THF, 40.0 mL, 55.0 mmol) was added to a solution of **18** (Supplementary data) (1.615 g, 10.21 mmol) in anhydrous THF (100 mL) stirring at −78 °C. This was stirred for 2 h and then allowed to warm to room temperature over 30 min. To the reaction mixture was added dropwise aqueous saturated aqueous NH_4_Cl (100 mL) and this was extracted with Et_2_O (3×100 mL). The combined organic phase was washed with saturated aqueous NaCl (100 mL), dried over anhydrous MgSO_4_, filtered and concentrated to give a yellow oil. Chromatography on silica gel with elution with 1:1 Et_2_O:CH_2_Cl_2_ containing 0.1% Et_3_N gave **19** as a pale yellow oil (1.312 g, 9.10 mmol, 89%). Analysis calcd for C_8_H_16_O_2_: C 66.6, H 11.2, found: C 66.8, H 11.1; TLC: R_f_ 0.14 (1:1 Et_2_O:CH_2_Cl_2_); HRMS (+ve ESI) *m/z* calcd for [M+Na]^+^: 167.1053, found: 167.1060; ^1^H NMR (500 MHz, CD_2_Cl_2_): *δ* (ppm) 1.23 (3H, s, **H8**), 1.32–1.40 (2H, m, **H5**), 1.46–1.55 (4H, m, **H4**, **H6**), 1.89 (2H, s (br), **H9**, **H10**), 3.57 (2H, t, *J*=6.3 Hz, **H7**), 5.01 (ABX system, 1H, dd, *J*_AX_=10.8 Hz, *J*_AB_=1.3 Hz, **H1a**), 5.17 (ABX system, 1H, dd, *J*_BX_=17.3 Hz, *J*_AB_=1.3 Hz, **H1b**), 5.90 (ABX system, 1H, dd, *J*_BX_=17.3 Hz, *J*_AX_=10.8 Hz, **H2**); ^13^C NMR (125 MHz, CD_2_Cl_2_): *δ* (ppm) 20.4 (**C5**), 27.9 (**C8**), 33.4 (**C6**), 42.2 (**C4**), 62.7 (**C7**), 73.4 (**C3**), 111.5 (**C1**), 145.8 (**C2**).

#### 4.2.6 2-(4-Hydroxybutyl)-2,5,7,8-tetramethyl-chromen-6-ol (**20**)

A solution of **19** (1.062 g, 7.37 mmol) and freshly prepared 2,3,5-trimethyl-*p*-hydroquinone (**10**, 1.031 g, 6.78 mmol) in formic acid (200 mL) were heated to reflux and refluxed for 3 h under an atmosphere of argon. The reaction was poured onto crushed ice (∼600 mL) and this was extracted with Et_2_O (3×200 mL) under argon. The combined organic phase was washed with H_2_O (3×200 mL) under argon, dried over anhydrous MgSO_4_ and concentrated. The oily brown residue was dissolved in MeOH (200 mL), conc. HCl (0.200 mL) was added and the reaction refluxed for a further 30 min under argon. The reaction was diluted with ice-cold H_2_O (400 mL) and this was extracted with Et_2_O (3×200 mL) under argon. The combined organic phase was washed under argon with H_2_O (3×200 mL), saturated aqueous Na_2_CO_3_ (3×200 mL) and H_2_O again (3×200 mL), dried over anhydrous MgSO_4_, filtered and concentrated to give a brown oil. Column chromatography on silica gel and elution with 1:9 Et_2_O:CH_2_Cl_2_ afforded **20** which recrystallised from CH_2_Cl_2_ to give a pale yellow solid (1.061 g, 3.81 mmol, 52%). Analysis calcd for C_17_H_26_O_3_: C 73.3, H 9.4, found: C 73.3, H 9.5; TLC: R_f_ 0.21 (1:9 Et_2_O:CH_2_Cl_2_), R_f_ 0.07 (1:4 EtOAc:petroleum ether 40–60); mp 102.7 °C; HRMS (+ve ESI) *m/z* calcd for [M+Na]^+^: 301.1774, found: 301.1769; ^1^H NMR (500 MHz, CD_2_Cl_2_): *δ* (ppm) 1.21 (3H, s, **H12**), 1.42–1.65 (6H, m, **H1′**–**H3′**), 1.72–1.82 (2H, m, **H3**), 2.065 (3H, s, **H11**), 2.073 (3H, s, **H9**), 2.12 (3H, s, **H10**), 2.58 (2H, t, *J*=7.0 Hz, **H4**), 3.59 (2H, t, *J*=6.3 Hz, **H4′**), 4.4 (1H s (br), **H1″**); ^13^C NMR (125 MHz, CD_2_Cl_2_): *δ* (ppm) 11.4 (**C9**), 11.9 (**C11**), 12.3 (**C10**), 20.3 (**C2′**), 21.0 (**C4**), 23.8 (**C12**), 32.0 (**C3**), 33.7 (**C3′**), 39.7 (**C1′**), 63.1 (**C4′**), 74.7 (**C2**), 117.8 (**C4a**), 118.9 (**C5**), 121.3, 122.7 (2C, 2× s, **C7**, **C8**), 145.0 (**C6**), 145.7 (**C8a**).

#### 4.2.7 4-(6-Methanesulfonyloxy-2,5,7,8-tetramethyl-chromen-2-yl)hexyl methanesulfonate (**21**)

A solution of **11** (0.323 g, 1.05 mmol) and Et_3_N (0.70 mL, 0.511 g, 5.05 mmol) was stirred in anhydrous CH_2_Cl_2_ (60 mL) at room temperature for 5 min. Methane sulfonyl chloride (MsCl) (0.20 mL, 0.136 g, 1.19 mmol) was added and the reaction was stirred for a further 1 h. The reaction mixture was washed with H_2_O (5×50 mL), and saturated aqueous NaHCO_3_ (50 mL), dried over anhydrous MgSO_4_, filtered and concentrated in vacuo to give a yellow oil. The crude oil was purified by crystallisation from EtOH to give **21** as a white solid (0.378 g, 0.82 mmol, 78%). Analysis calcd for C_15_H_28_O_3_: C 54.5, H 7.4, S 13.9, found: C 54.6, H 7.5, S 13.8; HRMS (+ve ESI) *m/z* calcd for [M+Na]^+^: 485.1638, found: 485.1633; ^1^H NMR (500 MHz, CDCl_3_): *δ* (ppm) 1.23 (3H, s, **H12**), 1.31–1.38 (2H, m, **H3′**), 1.38–1.47 (4H, m, **H2′**, **H4′**), 1.49–1.64 (2H, m, **H1′**), 1.71–1.84 (4H, m, **H3**, **H5′**), 2.09 (3H, s, **H11**), 2.21 (3H, s, **H9**), 2.24 (3H, s, **H10**), 2.59 (2H, t, *J*=6.8 Hz, **H4**), 2.99 (3H, s, **H7′**), 3.21 (3H, s, **H1″**), 4.21 (2H, t, *J*=6.5 Hz, **H6′**); ^13^C NMR (125 MHz, CDCl_3_): *δ* (ppm) 12.0 (**C11**), 13.7 (**C9**), 14.5 (**C10**), 20.7 (**C4**), 23.4 (**C2′**), 23.9 (**C12**), 25.4 (**C4′**), 29.2 (**C5′**), 29.5 (**C3′**), 31.1 (**C3**), 37.4 (**C7′**), 38.7 (**C1″**), 39.5 (**C1′**), 70.2 (**C6′**), 75.2 (**C2**), 118.1 (**C4a**), 123.8, 128.6 (2C, 2× s, **C7**, **C8**), 127.2 (**C5**), 139.7 (**C6**), 150.1 (**C8a**).

#### 4.2.8 (6-(6-Methanesulfonyloxy-2,5,7,8-tetramethyl-chromen-2-yl)hexyl)triphenylphosphonium methanesulfonate (**22**)

A mixture of triphenylphosphine (0.671 g, 2.56 mmol) and **21** (0.226 g, 0.49 mmol) in a Kimax tube was flushed with argon, sealed and stirred at 90 °C for 48 h. After cooling the crude product was dissolved in CH_2_Cl_2_ (0.5 mL) and precipitated from Et_2_O (20 mL) twice. The residual solvents were removed to give **22** as a sticky white solid (0.318 g, 0.44 mmol, 90%). HRMS (+ve ESI) *m/z* calcd for [M]^+^: 629.2849, found: 629.2862; ^1^H NMR (500 MHz, CDCl_3_): *δ* (ppm) 1.17 (3H, s, **H12**), 1.22–1.38 (4H, m, **H2′**, **H3′**), 1.41–1.54 (2H, m, **H1′**), 1.54–1.65 (4H, m, **H4′**, **H5′**), 1.67–1.78 (2H, m, **H3**), 2.01 (3H, s, **H11**), 2.17 (3H, s, **H9**), 2.20 (3H, s, **H10**), 2.54 (2H, t, *J*=6.8 Hz, **H4**), 2.69 (3H s, **H1‴**), 3.21 (3H s, **H1″**), 3.50–3.60 (2H, m, **H6′**), 7.64–7.72 (6H, m, **H9′**), 7.74–7.82 (9H, m, **H8′**, **H10′**); ^13^C NMR (125 MHz, CDCl_3_): *δ* (ppm) 11.9 (**C11**), 13.7 (**C9**), 14.5 (**C10**), 20.7 (**C4**), 21.9 (1C, d, *J*_CP_=50.0 Hz, **C6′**), 22.6 (1C, d, *J*_CCP_=4.3 Hz, **C5′**), 23.2, 29.6 (2C, 2× s, **C2′**, **C3′**), 24.0 (**C12**), 30.3 (1C, d, *J*_C3P_=16.0 Hz, **C4′**), 31.0 (**C3**), 38.7 (**C1″**), 39.2 (**C1′**), 39.5 (**C1‴**), 75.3 (**C2**), 118.1 (**C4a**), 118.6 (1C, d, *J*_CP_=85.0 Hz, **C7′**), 123.7, 128.6 (2C, 2× s, **C7**, **C8**), 127.2 (**C5**), 130.5 (6C, d, *J*_C3P_=12.3 Hz, **C9′**), 133.6 (6C, d, *J*_CCP_=9.8 Hz, **C8′**), 135.0 (3C, d, *J*_C4P_=2.9 Hz, **C10′**), 139.6 (**C6**), 150.1 (**C8a**); ^31^P NMR (121 MHz, CDCl_3_): *δ* (ppm) 25.6.

#### 4.2.9 4-(6-Methanesulfonyloxy-2,5,7,8-tetramethyl-chromen-2-yl)butyl methanesulfonate (**23**)

A solution of **20** (0.468 g, 1.68 mmol) and Et_3_N (1.12 mL, 0.818 g, 8.08 mmol) was stirred in anhydrous CH_2_Cl_2_ (30 mL) at room temperature for 5 min. MsCl (0.315 mL, 0.463 g, 4.04 mmol) was added and the solution was stirred for 1 h. The reaction mixture was washed with H_2_O (5×30 mL) and saturated aqueous NaHCO_3_ (30 mL), dried over anhydrous MgSO_4_, filtered and concentrated in vacuo to give a yellow solid which was recrystallised twice from EtOH to afford **23** as a white solid (0.543 g, 1.25 mmol, 74%). Analysis calcd for C_19_H_30_O_7_S_2_: C 52.5, H 7.0, S 14.8, found: C 52.5, H 7.3, S 14.6; TLC: *R*_*f*_ 0.69 (1:9 Et_2_O:CH_2_Cl_2_); HRMS (+ve ESI) *m/z* calcd for [M+Na]^+^: 457.1325, found: 457.1322; ^1^H NMR (500 MHz, CDCl_3_): *δ* (ppm) 1.25 (3H, s, **H12**), 1.52–1.68 (4H, m, **H1′**, **H2′**), 1.74–1.86 (4H, m, **H3**, **H3′**), 2.08 (3H, s, **H11**), 2.21 (3H, s, **H9**), 2.24 (3H, s, **H10**), 2.60 (2H, t, *J*=7 Hz, **H4**), 2.98 (3H, s, **H5′**), 3.23 (3H, s, **H1″**), 4.23 (2H, t, *J*=6.5 Hz, **H4′**); ^13^C NMR (125 MHz, CDCl_3_): *δ* (ppm) 12.0 (**C9**), 13.7 (**C11**), 14.5 (**C10**), 19.6 (**C2′**), 20.7 (**C4**), 23.9 (**C12**), 29.5 (**C3′**), 31.1 (**C3**), 37.4 (**C5′**), 38.8 (**C1″**), 39.0 (**C1′**), 69.9 (**C4′**), 75.0 (**C2**), 118.0 (**C4a**), 127.3 (**C5**), 123.8, 128.8 (2C, 2× s, **C7**, **C8**), 139.8 (**C6**), 150.0 (**C8a**).

#### 4.2.10 (4-(6-Methanesulfonyloxy-2,5,7,8-tetramethyl-chromen-2-yl)butyl)triphenyl -phosphonium methanesulfonate (**24**)

A mixture of **23** (0.314 g, 0.72 mmol) and triphenylphosphine (0.981 g, 3.74 mmol) was placed in a Kimax tube, flushed with argon, sealed and the reaction was stirred as a melt at 90 °C for 48 h. The cooled residue was dissolved in CH_2_Cl_2_ (∼2 mL) and precipitated from petroleum ether 40–60 (100 mL) three times. The residual solvents were removed in vacuo to give **24** as a white solid (0.464 g, 0.67 mmol, 92%). HRMS (+ve ESI) *m/z* calcd for [M]^+^: 601.2536, found: 601.2555; ^1^H NMR (500 MHz, CDCl_3_) *δ* (ppm) 1.21 (3H, s, **H12**), 1.52–1.59 (2H, m, **H1′**), 1.59–1.82 (6H, m, **H3**, **H2′**, **H3′**), 1.94 (3H, s, **H11**), 2.17 (3H, s, **H9**), 2.19 (3H, s, **H10**), 2.54 (2H, t, *J*=6.8 Hz, **H4**), 2.71 (3H, s, **H1‴**), 3.22 (3H, s, **H1″**), 3.48–3.70 (2H, m, **H4′**), 7.65–7.70 (6H, m, **H7′**), 7.74–7.80 (9H, m, **H6′**, **H8′**); ^13^C NMR (125 MHz) CDCl_3_: *δ* (ppm) 12.0 (**C11**), 13.7 (**C9**), 14.5 (**C10**), 20.6 (**C4**), 22.0 (1C, d, *J*_CP_=50.3 Hz, **C4′**), 23.1 (1C, d, *J*_CCP_=4.4 Hz, **C3′**), 24.0 (**C12**), 24.6 (1C, d, *J*_C3P_=16.1 Hz, **C2′**), 30.9 (**C3**), 38.7 (**C1″**), 38.8 (**C1′**), 39.6 (**C1‴**), 75.3 (**C2**), 118.2 (**C4a**), 118.5 (1C, d, *J*_CP_=85.6 Hz, **C5′**), 123.6, 128.6 (2C, 2× s, **C7**, **C8**), 127.3 (**C5**), 130.5 (1C, d, *J*_C3P_=12.5 Hz, **C7′**), 133.6 (1C, d, *J*_CCP_=9.9 Hz, **C6′**), 135.0 (1C, d, *J*_C4P_=2.9 Hz, **C8′**), 139.6 (**C6**), 149.9 (**C8a**); ^31^P NMR (121 MHz, CDCl_3_): *δ* (ppm) 25.5.

#### 4.2.11 2-(6-(Methanesulfonyloxy)-2,5,7,8-tetramethylchromen-2-yl)ethyl methanesulfonate (**25**)

A solution of **15** (0.013 g, 0.51 mmol) and Et_3_N (424 μL, 0.310 g, 3.06 mmol) was stirred in anhydrous CH_2_Cl_2_ (6 mL) at room temperature for 5 min. MsCl (88.0 μL, 0.129 g, 1.13 mmol) was added and the reaction was stirred for a further 1 h. The reaction mixture was diluted with CH_2_Cl_2_ (5 mL). This was washed with H_2_O (5×20 mL), dried over anhydrous MgSO_4_, filtered and concentrated in vacuo to give a white solid (0.224 g) which, after recrystallisation from EtOH to give **25** as white crystals (0.167 g, 0.41 mmol, 81%). Analysis calcd for C_17_H_26_O_7_S_2_: C 50.2, H 6.4, S; 15.8, found: C 50.3, H 6.6, S 15.7; TLC: R_f_ 0.76 (1:9 Et_2_O:CH_2_Cl_2_); mp 126.7 °C; HRMS (+ve ESI) *m/z* calcd for [M+Na]^+^: 429.1012, found: 429.1002; ^1^H NMR (500 MHz, CDCl_3_): *δ* (ppm) 1.31 (3H, s, **H12**), 1.86 (2H, td, *J*=7.0, 1.5 Hz, **H3**), 2.03–2.16 (2H, m, **H1′**), 2.09 (3H, s, **H11**), 2.21 (3H, s, **H9**), 2.24 (3H, s, **H10**), 2.63 (2H, t, *J*=7 Hz, **H4**), 3.00 (3H, s, **H3′**), 3.24 (3H, s, **H1″**), 4.38–4.52 (2H, m, **H2′**); ^13^C NMR (125 MHz, CDCl_3_): *δ* (ppm) 12.1 (**C10**), 13.7 (**C9**), 14.5 (**C11**), 20.5 (**C4**), 24.0 (**C12**), 31.5 (**C3**), 37.5 (**C3′**), 38.8(0) (**C1″**), 38.8(2) (**C1′**), 66.1 (**C2′**), 73.8 (**C2**), 117.7 (**C4a**), 123.9, 129.1 (2C, 2× s, **C7**, **C8**), 127.5 (**C5**), 140.0 (**C6**), 149.4 (**C8a**).

#### 4.2.12 (2-(6-(Methanesulfonyloxy)-2,5,7,8-tetramethyl-2*H*-chromen-2-yl)ethyl)triphenyl-phosphonium methanesulfonate (**26**)

A mixture of **25** (0.300 g, 0.74 mmol), NaI (0.558 g, 3.74 mmol) and triphenylphosphine (1.55 g, 3.69 mmol) was flushed with argon in a Kimax tube then sealed and the reaction was stirred as a melt at 90 °C for 48 h. The crude product was dissolved in CH_2_Cl_2_ (∼2 mL) and precipitated from petroleum ether 40–60 (100 mL) three times. The product was dissolved in methanol and passed down an anion exchange column loaded with ^−^OMs. The residual solvents were removed to give **26** as a white solid (0.450 g, 0.67 mmol, 91%). The UV spectrum of this compound has been reported previously.^23^ HRMS (+ve ESI) *m/z* calcd for [M]^+^: 573.2223, found: 573.2225; ^1^H NMR (500 MHz, CDCl_3_): *δ* (ppm) 1.45 (3H, s, **H12**), 1.88–1.08 (4H, m, **H3**, **H1′**), 2.01 (3H, s, **H11**), 2.12 (3H, s, **H9**), 2.22 (3H, s, **H10**), 2.40–2.60 (2H, m, **H4**), 2.66 (3H, s, **H1‴**), 3.25 (2H, s, **H1″**), 3.26–3.48, 3.92–4.04 (2H, 2× m, **H2′**), 7.61–7.67 (6H, m, **H5′**), 7.71–7.78 (9H, m, **H4′**, **H6′**), 7.71–7.78; ^13^C NMR (125 MHz, CDCl_3_): *δ* (ppm) 12.2 (**C11**), 13.6 (**C9**), 14.6 (**C10**), 17.4 (1C, d, *J*_CP_=53.4 Hz, **C2′**), 20.3 (**C4**), 23.9 (**C12**), 30.5 (**C3**), 31.0 (1C, d, *J*_CCP_=3.8 Hz, **C1′**), 38.8 (**C1″**), 39.5 (**C1‴**), 75.2 (1C, d, *J*_C3P_=13.9 Hz, **C2**), 118.1 (**C4a**), 118.3 (1C, d, *J*_CP_=86.1 Hz, **C15**), 123.3, 129.1 (1C, 2× s, **C7**, **C8**), 127.8 (**C5**), 130.5 (6C, d, *J*_C3P_=12.8, **C5′**), 133.6 (6C, d, *J*_CCP_=10.1 Hz, **C4′**), 135.1 (3C, d, *J*_C4P_=3.0 Hz, **C6′**), 139.8 (**C6**), 149.3 (**C8a**); ^31^P NMR (121 MHz, CDCl_3_): *δ* (ppm) 27.0.

#### 4.2.13 (2-(6-Hydroxy-2,5,7,8-tetramethyl-chromen-2-yl)ethyl)triphenylphosphonium methanesulfonate, MitoE_2_ (**1**)

A solution of lithium di*iso*propylamide was prepared by adding di*iso*propylamine (0.30 mL, 0.216 g, 2.13 mmol) to anhydrous THF (4 mL) at −78 °C followed by *n*-BuLi (1.8 M in hexane, 1.0 mL, 1.8 mmol). The solution was stirred at −78 °C for 30 min and then allowed to warm to 0 °C. The lithium di*iso*propylamide solution was then added to a solution of **26** (0.195 g, 0.29 mmol) in anhydrous THF (4 mL) with stirring at 0 °C. After 30 min the solution was allowed to warm to room temperature and then aqueous saturated NH_4_OMs (10 mL) was added. The aqueous layer was extracted with CH_2_Cl_2_ (3×10 mL) and the combined organic phases dried over anhydrous MgSO_4_, filtered and concentrated in vacuo to give a pale yellow oil (1.003 g). The crude product was dissolved in CH_2_Cl_2_ (0.5 mL) and precipitated from Et_2_O (20 mL) twice then chromatographed on silica gel with elution with 1:9 EtOH:CH_2_Cl_2_ and finally passed through an anion exchange column loaded with ^−^OMs in methanol. The residual solvents were removed by freeze drying to give **1** as a white solid (52.8 mg, 89.3 μmol, 31%). HRMS (+ve ESI) *m/z* calcd for [M]^+^: 495.2459, found: 495.2462; ^1^H NMR (500 MHz, CD_2_Cl_2_): *δ* (ppm) 1.37 (3H, s, **H12**), 1.87 (2H, t, *J*=7.0 Hz, **H3**), 1.69–1.78, 1.93–2.02 (2H, 2× m, **H1′**), 2.03 (3H, s, **H11**), 2.06 (3H, s, **H9**), 2.15 (3H, s, **H10**), 2.39–2.48, 2.53–2.62 (2H, 2× m, **H4**), 2.60 (3H, s, **H1‴**), 3.04–3.22, 3.28–3.46 (2H, 2× m, **H2′**), 3.70 (1H, s (br), **H1″**), 7.53 (6H, dd, *J*=12.7, 7.6 Hz, **H4′**), 7.63 (6H, td, *J*=7.7, 3.5 Hz, **H5′**), 7.83 (3H, t, *J*=7.4 Hz, **H6′**); ^13^C NMR (125 MHz, CD_2_Cl_2_): *δ* (ppm) 11.7 (**C9**), 12.0 (**C11**), 12.7 (**C10**), 17.9 (1C, d, *J*_CP_=54.8 Hz, **C2′**), 20.7 (**C4**), 24.4 (**C12**), 29.9 (**C1′**), 32.0 (**C3**), 39.7 (3C, s, **C1‴**), 73.7 (1C, d, *J*_C3P_=13.5 Hz, **C2**), 117.4 (**C4a**), 118.1 (1C, d, *J*_CP_=86.4 Hz, **C3′**), 120.7 (**C5**), 122.0, 123.0 (1C, 2× s, **C7**, **C8**), 130.9 (6C, d, *J*_C3P_=12.9, **C5′**), 133.6 (6C, d, *J*_CCP_=9.8 Hz, **C4′**), 135.8 (3C, d, *J*_C4P_=3.1 Hz, **C6′**), 144.2 (**C8a**), 146.6 (**C6**); ^31^P NMR (121 MHz, CD_2_Cl_2_): *δ* (ppm) 26.1.

#### 4.2.14 (4-(6-Hydroxy-2,5,7,8-tetramethyl-chroman-2-yl)-butyl)triphenylphosphonium methanesulfonate, MitoE_4_ (**2**)

A solution of lithium di*iso*propylamide (2.07 mmol) in anhydrous THF (4.0 mL) was prepared as for **2** above and added to a solution of **24** (0.192 g, 0.28 mmol) in anhydrous THF (4.0 mL) stirring at 0 °C. After 30 min the solution was allowed to warm up to room temperature and then aqueous saturated NH_4_OMs (10 mL) was added. The aqueous layer was extracted with CH_2_Cl_2_ (3×10 mL). The combined organic phases were dried over anhydrous MgSO_4_, filtered and concentrated in vacuo to give a pale yellow oil. The crude product was dissolved in CH_2_Cl_2_ (0.5 mL) and precipitated from Et_2_O (20 mL) twice and then purified by column chromatography on silica gel and elution with 1:9 EtOH:CH_2_Cl_2_ to afford **2** as a white solid (0.091 g, 0.15 mmol, 53%). TLC: *R*_*f*_ 0.22 (1:9 EtOH:CH_2_Cl_2_); HRMS (+ve ESI) *m/z* calcd for [M]^+^: 523.2760, found: 523.2771; ^1^H NMR (500 MHz, CD_2_Cl_2_) *δ* (ppm) 1.18 (3H, s, **H12**), 1.42–1.66 (2H, m, **H1′**), 1.66–1.79 (6H, m, **H3**, **H2′**, **H3′**), 1.94 (3H, s, **H11**), 2.07 (3H, s, **H9**), 2.08 (3H, s, **H10**), 2.55 (2H, t, *J*=6.8 Hz, **H4**), 2.61 (3H, s, **H1‴**), 3.14–3.24 (2H, m, **H4′**), 3.47 (1H, s, **H1″**), 7.62–7.72 (12H, m, **H6′**, **H7′**), 7.84 (3H, t *J*=7.3 Hz, **H8′**); ^13^C NMR (125 MHz) CD_2_Cl_2_: *δ* (ppm) 11.6 (**C9**), 11.9 (**C11**), 12.5 (**C10**), 21.0 (**C4**), 23.0 (1C, d, *J*_CP_=50.6 Hz, **C4′**), 23.2 (**C3′**), 24.1 (**C12**) 25.1 (1C, d, *J*_C3P_=15.9 Hz, **C2′**), 32.1 (**C3**), 38.2 (**C1′**), 39.6 (**C1‴**), 74.3 (**C2**), 117.6 (**C4a**), 118.3 (1C, d, *J*_CP_=85.6 Hz, **C5′**), 119.7 (**C5**), 122.0, 122.2 (2C, 2× s, **C7**, **C8**), 130.9 (6C, d, *J*_C3P_=12.5 Hz, **C7′**), 133.8 (6C, d, *J*_CCP_=9.8 Hz, **C6′**), 135.7 (3C, d, *J*_C4P_=2.6 Hz, **C8′**), 145.2 (**C6**), 145.5 (**C8a**); ^31^P NMR (121 MHz, CD_2_Cl_2_): *δ* (ppm) 24.8.

#### 4.2.15 (6-(6-Hydroxy-2,5,7,8-tetramethyl-chroman-2-yl)-hexyl)triphenyl phosphonium methanesulfonate, MitoE_6_ (**3**)

A solution of lithium di*iso*propylamide (1.49 mmol) in anhydrous THF (5.0 mL) was prepared as for **2** above and added to a solution of **22** (0.152 g, 0.21 mmol) in anhydrous THF (10 mL) with stirring at 0 °C. After 30 min the solution was allowed to warm to room temperature and then aqueous saturated NH_4_OMs (10 mL) was added. The aqueous layer was extracted with CH_2_Cl_2_ (3×1 mL). The combined organic phases were dried over anhydrous MgSO_4_, filtered and concentrated in vacuo to give a pale yellow oil which was dissolved in CH_2_Cl_2_ (0.5 mL) and precipitated from Et_2_O (20 mL) twice. Purification by column chromatography on silica gel and elution with 1:9 EtOH:CH_2_Cl_2_ followed by freeze drying gave **3** as a white solid (0.067 g, 0.10 mmol, 49%). TLC: *R*_*f*_ 0.19 (1:9 EtOH:CH_2_Cl_2_); HRMS (+ve ESI) *m/z* calcd for [M]^+^: 551.3073, found: 551.3078; ^1^H NMR (500 MHz, CD_2_Cl_2_): *δ* (ppm) 1.17 (3H, s, **H12**), 1.24–1.27 (4H, m, **H2′**, **H3′**), 1.42–1.57 (4H, m, **H1′**, **H4′**), 1.57–1.66 (2H, m, **H5′**), 1.66–1.80 (2H, m, **H3**), 2.00 (3H, s, **H11**), 2.07 (3H, s, **H9**), 2.10 (3H, s, **H10**), 2.50 (3H s, **H1‴**), 2.55 (2H, t, *J*=6.9 Hz, **H4**), 3.22–3.30 (2H, m, **H6′**), 7.66–7.77 (12H, m, **H8′**, **H9′**), 7.82–7.86 (3H, m, **H10′**); ^13^C NMR (125 MHz, CD_2_Cl_2_): *δ* (ppm) 11.6 (**C9**), 11.9 (**C11**), 12.8 (**C10**), 21.0 (**C4**), 22.86 (1C, d, *J*_CCP_=4.5 Hz, **C5′**), 22.9 (1C, d, *J*_CP_=50.9 Hz, **C6′**), 23.5 (**C2′**), 24.0 (**C12**), 29.7 (**C3′**), 30.8 (1C, d, *J*_C3P_=16.1 Hz, **C4′**), 30.1 (**C3**), 39.7 (**C1′**), 39.0 (**C1‴**), 75.6 (**C2**), 117.7 (**C4a**), 118.3 (1C, d, *J*_CP_=85.5 Hz, **C7′**), 119.5 (**C5**), 121.8, 122.4 (2C, 2× s, **C7**, **C8**), 130.9 (6C, d, *J*_C3P_=12.6 Hz, **C9′**), 133.9 (6C, d, *J*_CCP_=9.9 Hz, **C8′**), 135.6 (3C, d, *J*_C4P_=3.3 Hz, **C10′**), 145.3 (**C6**), 145.6 (**C8a**); ^31^P NMR (121 MHz, CD_2_Cl_2_): *δ* (ppm) 24.8.

#### 4.2.16 (10-(6-Hydroxy-2,5,7,8-tetramethyl-chroman-2-yl)-decyl)triphenylphosphonium methanesulfonate, MitoE_10_ (**4**)

A mixture of 10-(3,4-dihydro-6-hydroxy-2,5,7,8-tetramethyl-chromen-2-yl)decyl methanesulfonate (S7, Supplementary data) (0.095 g, 0.21 mmol) and triphenylphosphine (0.282 g, 1.07 mmol) was mixed in a Kimax tube then the tube was flushed with argon, sealed and stirred at 80 °C for 24 h. The tube was then cooled and crude product dissolved in CH_2_Cl_2_ (0.5 mL) and precipitated twice from Et_2_O to give a pale yellow oil (0.027 g). The oil was dissolved in EtOH (0.5 mL) and then diluted with H_2_O (5 mL) and any volatile solvents were removed in vacuo. The aqueous solution was freeze dried to give **4** as a fluffy white solid (27.0 mg, 37.4 μmol, 18%). HRMS (+ve ESI) *m/z* calcd for [M]^+^: 607.3699, found: 607.3687; ^1^H NMR (500 MHz, CD_2_Cl_2_) *δ* (ppm) 1.26–1.30 (8H, m, **H3′**–**H6′**) 1.20 (3H, s, **H12**), 1.34–1.46 (2H, m, **H2′**), 1.46–1.57 (4H, **H1′**, **H7′**), 1.57–1.67 (4H, m, **H8′**, **H9′**), 1.69–1.81 (2H, m, **H3**), 2.04 (3H, s, **H11**), 2.06 (3H, s, **H9**), 2.10 (3H, s, **H10**), 2.53 (3H, s, **H1‴**), 2.56 (2H, t, *J*=6.8 Hz, **H4**), 3.25–3.31 (2H, m, **H10′**), 7.68–7.73 (12H, m, **H12′**, **H13′**), 7.81–7.85 (3H, m, **H14′**); ^13^C NMR (125 MHz) CD_2_Cl_2_: *δ* (ppm) 11.6 (**C9**) 11.9 (**C11**), 12.6 (**C10**), 21.1 (**C4**), 22.8 (1C, d, *J*_CP_=50.4 Hz, **C10′**), 22.8 (1C, d, *J*_CCP_=4.5 Hz, **C9′**), 23.6 (**C2′**) 24.2 (**C12**), 29.2, 29.5, 30.2 (3C, 5× s, **C3′**–**C7′**), 30.9 (1C, d, *J*_C3P_=16.0 Hz, **C8′**), 32.2 (**C3**), 38.8 (**C1′**), 39.7 (**H1‴**), 74.6 (**C2**), 117.7 (**C4a**), 118.5 (1C, d, *J*_CP_=85.5 Hz, **C11′**), 119.5 (**C5**), 121.9, 122.4 (2C, 2× s, **C7**, **C8**), 130.8 (1C, d, *J*_C3P_=12.5 Hz, **C13′**), 133.9 (1C, d, *J*_CCP_=10.1 Hz, **C12′**), 135.5 (1C, d, *J*_C4P_=2.8 Hz, **C14′**), 145.4 (**C6**), 145.5 (**C8a**); ^31^P NMR (121 MHz) CD_2_Cl_2_: *δ* (ppm) 24.9.

#### 4.2.17 (11-(6-Hydroxy-2,5,7,8-tetramethyl-chroman-2-yl)-undecyl)triphenylphosphonium methanesulfonate, MitoE_11_ (**5**)

A mixture of 11-(6-Hydroxy-2,5,7,8-tetramethyl-chromen-2-yl)undecyl methanesulfonate (S11, Supplementary data) (0.097 g, 0.21 mmol) and triphenylphosphine (0.285 g, 1.09 mmol) was placed in a Kimax tube, which was flushed with argon, sealed and stirred at 80 °C for 48 h. The cooled reaction mixture was dissolved in CH_2_Cl_2_ (0.5 mL) and precipitated twice from Et_2_O to give **5** as a pale yellow oil (0.087 g, 0.12 mmol, 57%). LRMS (+ve ESI) calcd for [M]^+^: 621, found 621; HRMS (+ve ESI) calcd for [M]^+^: 621.3856, found 621.3853; ^1^H NMR (500 MHz, CDCl_3_) *δ* (ppm) 1.15 (12H, s (br), **H3′**–**H8′**) 1.20 (3H, s, **H12**), 1.36 (2H, quin, *J*=7.3 Hz, **H2′**), 1.42–1.58 (2H, m, **H1′**), 1.52–1.60 (4H, m, **H9′, H10′**), 1.69–1.81 (2H, m, **H3**), 2.07 (3H, s, **H11**), 2.09 (3H, s, **H9**), 2.12 (3H, s, **H10**), 2.56 (2H, t, *J*=6.8 Hz, **H4**), 2.69 (3H, s, **H1‴**), 3.53 (2H, m, **H11′**), 7.66–7.72 (6H, m, **H14′**), 7.74–7.81 (9H, m, **H13′**, **H15′**); ^13^C NMR (125 MHz) CDCl_3_: *δ* (ppm) 11.5 (**C9**) 11.8 (**C11**), 12.4 (**C10**), 20.8 (**C4**), 21.5 (1C, d, *J*_CP_=50.0 Hz, **C11′**), 22.7 (1C, d, *J*_CCP_=4.5 Hz, **C10′**), 23.5 (**C2′**) 24.0 (**C12**), 29.2, 29.3(9), 29.4(1), 29.5, 30.0 (6C, 5× s, **C3′**–**C8′**), 30.4 (1C, d, *J*_C3P_=15.6 Hz, **C9′**), 31.7 (**C3**), 39.0 (**C1′**), 39.5 (**H1‴**), 74.4 (**C2**), 117.3 (**C4a**), 118.6 (1C, d, *J*_CP_=85.6 Hz, **C12′**), 119.0 (**C5**), 121.5, 122.4 (2C, 2× s, **C7**, **C8**), 144.9 (**C6**), 145.4 (**C8a**), 130.5 (1C, d, *J*_C3P_=12.3 Hz, **C14′**), 133.6 (1C, d, *J*_CCP_=9.8 Hz, **C13′**), 135.0 (1C, d, *J*_C4P_=2.9 Hz, **C15′**); ^31^P NMR (121 MHz) CDCl_3_: *δ* (ppm) 25.4.

### 4.3 Measurement of lipid peroxidation rat brain homogenates

Rat brain homogenates were prepared and incubated for 30 min at 37 °C as described^23^ with a range of concentration of MitoE compounds, Trolox or ethanol carrier. The extent of lipid peroxidation was assessed by measuring TBARS.^22^ Values were corrected for the background level at *t*=0 and were expressed as a % of the control with EtOH carrier. Data are the means±SD of 4–6 independent experiments.

### 4.4 Mitochondrial and cell experiments

Rat liver mitochondria were prepared as described.^22^ Mitochondrial uptake of MitoE_2_, MitoE_10_ was measured using an ion-selective electrode for TPP, as previously described^21^ Briefly, isolated rat liver mitochondria (1 mg protein/mL) were incubated in 120 mM KCl, 10 mM HEPES, 1 mM EGTA, pH 7.2, KOH supplemented with 4 μg/mL rotenone at 37 °C with stirring. The TPP-electrode response was calibrated with five successive additions of 1 μM MitoE_2_ or MitoE_10_ (from a 1 mM stock in EtOH). The mitochondria were energised with 10 mM succinate as a respiratory substrate to induce membrane potential-dependent uptake of the MitoE compound into the mitochondrial matrix. Finally the mitochondria were uncoupled with 1 μM FCCP, dissipating the membrane potential and resulting in release of MitoE.

To measure mitochondrial lipid peroxidation, isolated rat liver mitochondria (2 mg protein/mL) were incubated in KCl medium as above, supplemented with 10 mM succinate and 4 μg/mL rotenone at 37 °C and pretreated±MitoE for 2 min. Lipid peroxidation was then initiated with 1 mM cumene hydroperoxide, and the mitochondria were incubated for a further 15 min. Lipid peroxidation was assayed as TBARS as above. Values were corrected for background level without cumene hydroperoxide and were expressed as a % of the control with EtOH carrier. Data are the means±SD of 3–4 independent experiments.

To measure mitochondrial DNA damage, C2C12 cells were seeded at 20,000 cells/cm^2^ in six-well culture plates and grown overnight at 37 °C. Cells were then pre-incubated in 100 nM MitoE_10_ or *n*-decyltriphenylphosphonium bromide (decylTPP) for 30 min, and then 25 μM menadione was added to generate oxidative damage within the cells and incubated for a further 1 h. Cells were washed and DNA was isolated using the DNeasy Blood and Tissue kit from Qiagen, quantitated using the Picogreen Assay (Invitrogen), and then the extent of oxidative damage was assessed by a quantitative PCR assay comparing the relative amplification of a long (∼10 kbp) of mitochondrial DNA normalised to a short (∼100 bp) section as described. Data are expressed relative to untreated cells and are means±SEM of three independent experiments. Statistical significance was determined by Student's *t*-test. **p<0.01 compared against menadione treated samples.

## Figures and Tables

**Table 1:** Selected ^1^H NMR resonances in the MitoE mesylate series

Assignment	MitoE_2_	MitoE_4_	MitoE_6_	MitoE_10_
*δ*^a^	*δ*^a^	*δ*^a^	*δ*^a^
4	2.44^b^, 2.58^b^	2.55^c^	2.55^c^	2.56^c^
9	2.06	2.07	2.07	2.06
10	2.15	2.08	2.10	2.10
11	2.03	1.94	2.00	2.04
12	1.37	1.18	1.17	1.20
1′	1.74^b^, 1.98^b^	1.54^b^	1.50^b^	1.52^b^
n′^d^	3.13^b^, 3.37^b^	3.19^b^	3.26^b^	3.28^b^
^−^OMs	2.60	2.66	2.50	2.53

a Chemical shift in CD_2_Cl_2_ referenced to solvent (5.31 ppm).

b Value of *δ* at centre of multiplet.

c Triplet (*J*=6.8 Hz).

d Methylene adjacent to P^+^.

## Footnotes

Supplementary data associated with this article can be found in the online version, at http://dx.doi.org/10.1016/j.tet.2015.09.014.
