# Supplementary material for: Synthesis of triphenylphosphonium vitamin E derivatives as mitochondria-targeted antioxidants
Source: Tetrahedron. 2015 Nov 4;71(44):8444–53. doi: 10.1016/j.tet.2015.09.014 (PMC4596152; doi:10.1016/j.tet.2015.09.014)
Supplement: Supplementary file 1 [file mmc1.docx]

**Supplementary Data**

#### 2-(4,8,12-Trimethyltridecyl)-2,5,7,8-tetramethyl-chroman-6-yl methanesulfonate (vitamin E mesylate)

A solution of vitamin E (Aldrich, α-tocopherol, 0.496 g, 1.15 mmol) and Et_3_N (0.32 mL, 0.235 g, 2.32 mmol) was stirred in anhydrous CH_2_Cl_2_ (10 mL) at room temperature for 5 min. MsCl (0.100 mL, 0.147 g, 1.28 mmol) was added and the reaction was stirred for a further 1 h. The reaction mixture was diluted with CH_2_Cl_2_ (10 mL). This was washed with H_2_O (3 × 20 mL), dried over anhydrous MgSO_4_, filtered and concentrated *in vacuo* to give a yellow oil (0.586 g) which was purified by column chromatography on silica gel. Elution with 1:4 Et_2_O:petroleum ether gave the product (vitamin E mesylate) as a colourless gum (0.563 g, 1.11 mmol, 96%).

Analysis calcd. for C_30_H_52_O_4_S: C, 70.82, H, 10.30, S, 6.30, found: C, 71.04, H 10.49, S 6.10; LRMS (+ve APCI) calcd. for [M+H]^+^: 509, found 509; ^1^H NMR (500 MHz, CDCl_3_) δ (ppm): 0.83 – 0.90 (12H, m, **H13’** – **H16’**), 1.02 – 1.64 (21H, m, **H1’** – **H12’**), 1.24 (3H, s, **H12**), 1.74 – 1.86 (2H, m, **H3**), 2.10 (3H, s, **H11**), 2.21 (3H, s, **H9**), 2.24 (3H, s, **H10**), 2.59 (2H, t, *J* = 6.9 Hz, **H4**), 3.23 (3H, s, **H1’’**); ^13^C NMR (125 MHz, CDCl_3_) δ (ppm): 12.0 (**C11**), 13.7 (**C9**), 14.5 (**C10**), 19.8, 19.8 (2C, 2 × s, **C15’**, **C16’**), 20.8 (**C12**), 22.7, 22.8 (2C, 2 × s, **C13’**, **C14’**), 21.1 (**C2’**), 24.5, 24.9 37.4, 37.4(5), 37.4(7), 37.5(2), 39.4 (7C, 7 s, **C3’**, **C5’** – **C7’**, **C9’** – **C11’**), 24.0 (**C12**), 28.0 (**C12’**), 30.9(9), 31.0(4) (1C, 2 s, **C3**), 31.7(5), 32.7(7), 32.8(4), 32.8(6) (2C, 4 s, **C4’**, **C8’**), 38.7 (**C1’’**), 40.0(8), 40.1(3) (**C1’**), 75.4 (**C2**), 118.1 (**C4a**), 123.7, 128.6 (2C, 2 s, **C7**, **C8**), 127.2 (**C5**), 139.7 (**C6**), 150.3 (**C8a**).

**Demesylation of vitamin E mesylate**

A solution of lithium di*iso*propylamide was prepared by adding di*iso*propylamine (1.26 mL, 0.907 g, 8.97 mmol) to anhydrous THF (12.0 mL) at -78 ºC followed by *n*-BuLi (1.8 M in hexane, 4.20 mL, 7.56 mmol). The solution was stirred at -78ºC for 30 min and then allowed to warm to 0ºC. The lithium di*iso*propylamide was added to a solution of vitamin E mesylate (1.277 g, 2.51 mmol) in anhydrous THF (25 mL) stirring at 0ºC. After 30 min the solution was allowed to warm up to room temperature and then saturated aqueous NH_4_Cl (50 mL) was added. The aqueous layer was extracted with CH_2_Cl_2_ (3 × 50 mL) and the combined organic phases were washed with saturated aqueous NaCl (50 mL), dried over MgSO_4_, filtered and concentrated *in vacuo* to give a light brown oil (1.00 g) whose ^1^H NMR spectrum was in complete agreement with that of the Viatmin E used to prepare the reactant.

Scheme for the synthesis of MitoE_10_ precursor **S7:**

#### 12-Hydroxy-dodec-2-one (S1)

A solution of Hg(OTf)_2_.(TMU)_2_ was prepared by stirring HgO (0.129 g, 0.60 mmol) and Tf_2_O (100 μL, 0.596 g, 0.21 mmol) in CH_3_CN (8 mL), stirred until it went colourless (~10 min), TMU (140 μL, 0.144 g, 1.24 mmol) was added and the solution was stirred for a further 5 min. To this was added H_2_O (1.27 mL, 1.27 g, 70.05 mmol) and then CH_2_Cl_2_ (3.20 mL). A solution of 11-dodecyn-1-ol (2.137 g, 11.72 mmol) in a mixture of CH_3_CN (1.20 mL) and CH_2_Cl_2_ (0.48 mL) was prepared. This was added to the solution of Hg(OTf)_2_.(TMU)_2_ over 5 min and the reaction was left to stir for 48 h. The reaction was poured into saturated aqueous NaCl:NaHCO_3_ 1:1 (50 mL) and this was extracted with Et_2_O (3 × 50 mL). The combined organic phase was dried over anhydrous MgSO_4_, filtered and concentrated to give a yellow oil. The crude product was crystallised from pentane to afford **S1** as a shiny white solid (1.817 g, 9.07 mmol, 81 %). ^1^H NMR data were consistent with the literature.^1^

#### 12-(Tetrahydro-*2H*-pyran-2-yloxy)-dodecan-2-one (S2)

A solution of **S1** (1.793 g, 8.95 mmol), DHP (1.22 mL, 1.132 g, 1.35 mmol) and PPTS (0.224 g, 0.89 mmol) in anhydrous CH_2_Cl_2_ (12 mL) was stirred at room temperature for 4 h. The solvents were removed and the residue was dissolved in Et_2_O (20 mL). This was washed with saturated aqueous NaCl (3 × 20 mL), dried over anhydrous MgSO_4_, filtered and concentrated to give **S2** as a pale yellow oil (0.499 g, 1.75 mmol, 96 %). ^1^H NMR data were consistent with the literature^1^ and the crude product was used without further purification.

#### 13-(Tetrahydro-2H-pyran-2-yloxy)-3-hydroxy-3-methyl-tridec-1-ene (S3)

Vinylmagnesium bromide (1.0 M in THF, 4.0 mL, 4.0 mmol) was added to a solution of **S2** (0.470 g, 1.65 mmol) in anhydrous THF (15 mL) stirring at -78ºC. This was stirred for 3 h and then allowed to warm to room temperature over 30 min. The reaction mixture was added dropwise to saturated aqueous NH_4_Cl (50 mL) and extracted with Et_2_O (3 × 50 mL). The combined organic phase was washed with saturated aqueous NaCl (3 × 50 mL), dried over anhydrous MgSO_4_, filtered and concentrated to give a pale yellow oil (0.562 g). The crude reaction product was chromatographed on silica gel. Elution with 1:4 Et_2_O:petroleum ether 40-60 containing 0.1% Et_3_N gave **S3** as a colourless oil (0.4916 g, 1.57 mmol, 95%). Analysis calcd. for C_19_H_35_O_3_: C 73.03, H 11.61, found: C 72.80, H 11.74; TLC: R_f_ 0.51 (1:1 Et_2_O:petroleum ether 40-60); LRMS (+ve APCI) *m/z* calcd. for [M-OH]^+^: 295, found 295, *m/z* calcd. for [M+H]^+^: 313, found 313, *m/z* calcd. for [M+Na]^+^: 335, found 335; ^1^H NMR (500 MHz, CD_2_Cl_2_): δ (ppm) 1.22 (3H, s, **H14**), 1.24 – 1.38 (14H, m, **H5** – **H11**), 1.42 – 1.58 (8H, m, **H4**, **H12**, **H16a**, **H17a**, **H18**), 1.62 – 1.70 (1H, m, **H16b**), 1.74 – 1.83 (1H, m, **H17b**), 3.33 (1H, dt, *J*_HCH_ = 9.6, *J*_HCCH_ = 6.7 Hz, **H13a**), 3.41 – 3.47 (1H, m, **H19a**), 3.67 (1H, dt, *J*_HCCH_ = 9.6, *J*_HCH_ = 6.7 Hz, **H13b**), 3.81 (1H, ddd, *J* = 11.1, 8.4, 3.3 Hz, **H19b**), 4.53 (1H, t, *J* = 3.8 Hz, **H15**), 5.00 (ABX system, 1H, dd, *J*_AX_ = 10.8 Hz, *J*_AB_ = 1.4 Hz, **H1a**), 5.16 (ABX system, 1H, dd, *J*_BX_ = 17.5 Hz, *J*_AB_ = 1.4 Hz, **H1b**), 5.90 (ABX system, 1H, dd, *J*_BX_ = 17.5 Hz, *J*_AX_ = 10.8 Hz, **H2**); ^13^C NMR (125 MHz, CD_2_Cl_2_): δ (ppm) 20.0 (**C17**), 24.3 (**C5**), 26.0 (**C18**), 26.6 (**C11**), 27.9 (**C2’**), 29.8, 29.9, 30.0, 30.4 (5C, 4 × s (br), **C6** – **C10**), 30.1 (**C12**), 31.2 (**C16**), 42.8 (**C4**), 62.4 (**C19**), 67.8 (**C13**), 73.4 (**C3**), 99.1 (**C15**), 111.3 (**C1**), 146.0 (**C2**).

#### 2-(10-Hydroxydecyl)-2,5,7,8-tetramethyl-chromen-6-ol (S4)

A solution of **S3** (1.261 g, 3.89 mmol) and freshly prepared 2,3,5-trimethyl-*p*-hydroquinone (**10**, 0.412 g, 2.71 mmol) in formic acid (60 mL) were heated to reflux and refluxed for 4 h under an atmosphere of argon. The reaction was poured into crushed ice (~ 180 mL) and this was extracted with Et_2_O (3 × 100 mL) under argon. The combined organic phase was washed with H_2_O (3 × 100 mL) under argon, dried over anhydrous MgSO_4_ and concentrated. The oily brown residue was dissolved in MeOH (60 mL), conc. HCl (0.060 mL) was added and the reaction refluxed for a further 30 min under argon. The reaction was diluted with ice cold H_2_O (100 mL) and this was extracted with Et_2_O (3 × 100 mL) under argon. The combined organic phase was washed under argon with H_2_O (2 × 100 mL), saturated aqueous Na_2_CO_3_ (3 × 100 mL) and H_2_O again (2 × 100 mL), dried over anhydrous MgSO_4_, filtered and concentrated to give a brown oil. The crude product was purified by column chromatography on silica gel. The product was eluted with CH_2_Cl_2_:Et_2_O 9:1 and **S4** was obtained as a pale yellow solid (0.527 g, 1.45 mmol, 54%). This is a known compound^2^ but no analysis has been reported in the literature. Analysis calcd. for C_23_H_38_O_3_: C 76.20, H 10.56, found: C 76.02 H 10.54; TLC: R_f_ 0.41 (1:3 Et_2_O:CH_2_Cl_2_); m.p. 90.0ºC; LRMS (-ve ESI) *m/z* calcd. for [M-H]^-^: 361, found: 361; ^1^H NMR (500 MHz, CD_2_Cl_2_) δ (ppm) 1.20 (3H, s, **H12**), 1.24 – 1.35 (12H, m, **H3’** – **H8’**), 1.35 – 1.44 (2H, m, **H2’**), 1.45 – 1.60 (4H, m, **H1’**, **H9’**), 1.70 – 1.82 (2H, m, **H3**), 2.06 (3H, s, **H11**), 2.07 (3H, s, **H9**), 2.12 (3H, s, **H10**), 2.58 (2H, t, *J* = 7.0 Hz, **H4**), 3.57 (2H, t, *J* = 6.5 Hz, **H10’**); ^13^C NMR (125 MHz, CD_2_Cl_2_): δ (ppm) 11.4 (**C9**), 11.8 (**C11**), 12.3 (**C10**), 20.1 (**C4**), 23.9(1) (**C12**), 23.9(3) (**C2’**), 26.1 (**C8’**), 29.8, 29.8(7), 29.9(3), 30.0, 30.5 (5C, 5 × s, **C3’** – **C7’**), 32.0 (**C3**), 33.2 (**C9’**), 39.6 (**C1’**), 63.2 (**C10’**), 74.8 (**C2**), 117.8 (**C4a**), 118.9 (**C5**), 121.3, 122.7 (2C, 2 × s, **C7**, **C8**), 144.9 (**C6**), 145.8 (**C8a**).

#### 10-(6-Benzyloxy-2,5,7,8-tetramethyl-chromen-2-yl)decan-1-ol (S5)

BnBr (320 μL, 0.461 g, 2.69 mmol) and anhydrous Cs_2_CO_3_ (0.885 g, 2.72 mmol) were added to a solution of **S4** (0.328 g, 0.91 mmol) in anhydrous CH_3_CN (10 mL). The reaction was heated to reflux and refluxed for 24 h. The reaction was diluted with H_2_O (20 mL) and this was extracted with Et_2_O (3 × 20 mL). The combined organic phase was washed with saturated aqueous NaCl (20 mL), dried over anhydrous MgSO_4_, filtered and concentrated *in vacuo* to give an orange oil. The crude product was purified by column chromatography on silica gel. Elution with 1:9 Et_2_O:hexane gave **S5** as a pale yellow solid (0.262 g, 0.58 mmol, 64%). HRMS (+ve ESI) *m/z* calcd. for [M+Na]^+^: 475.3183, found: 475.3161; ^1^H NMR (300 MHz, CDCl_3_): δ (ppm) 1.20 (3H, s, **H12**), 1.24 – 1.44 (12H, m, **H2’** – **H8’**), 1.45 – 1.64 (4H, m, **H1’**, **H9’**), 1.70 – 1.89 (2H, m, **H3**), 2.10 (3H, s, **H11**), 2.17 (3H, s, **H9**), 2.22 (3H, s, **H10**), 2.59 (2H, t, *J* = 6.8 Hz, **H4**), 3.64 (2H, t, *J* = 6.6 Hz, **H10’**), 4.70 (2H, s, **H1’’**), 7.27 – 7.42 (3H, m, **H4’’**, **H5’’**), 7.50 (2H, d, *J* = 7.5 Hz, **H3’’**).

#### 10-(6-Benzyloxy-2,5,7,8-tetramethyl-chromen-2-yl)decyl methanesulfonate (S6)

A solution of **S5** (0.262 g, 0.58 mmol) and Et_3_N (0.150 mL, 0.110 g, 1.08 mmol) was stirred in anhydrous CH_2_Cl_2_ (5 mL) at room temperature for 5 min. MsCl (0.050 mL, 0.074 g, 0.64 mmol) was added and the reaction was stirred for a further 90 min. The reaction mixture was diluted with CH_2_Cl_2_ (10 mL). This was washed with H_2_O (3 × 20 mL), dried over anhydrous MgSO_4_, filtered and concentrated *in vacuo* to give a yellow oil (0.278 g). The crude product was purified by column chromatography on silica gel. Elution with 1:3 Et_2_O:petroleum ether 40-60 afforded pure **S6** as a pale yellow solid (0.254 g, 0.46 mmol, 80%). ^1^H NMR (500 MHz, CDCl_3_): δ (ppm) 1.24 (3H, s, **H12**), 1.25 – 1.35 (10H, m, **H3’** – **H7’**), 1.35 – 1.45 (4H, m, **H2’**, **H8’)**, 1.50 – 1.64 (2H, m, **H1’**), 1.71 – 1.86 (4H, m, **H3**, **H9’**) 2.10 (3H, s, **H11**), 2.17 (3H, s, **H9**), 2.22 (3H, s, **H10**), 2.59 (2H, t, *J* = 6.8 Hz, **H4**), 3.00 (3H, s, **H11’**), 4.22 (2H, t, *J* = 6.5 Hz, **H10’**), 4.70 (2H, s, **H1’’**), 7.34 (1H, t, *J* = 7.5 Hz, **H5’’**), 7.39 (2H, t, *J* = 7.5 Hz, **H4’’**), 7.50 (2H, d, *J* = 7.5 Hz, **H3’’**); ^13^C NMR (125 MHz, CDCl_3_): δ (ppm) 11.9 (**C11**), 12.1 (**C9**), 12.9 (**C10**), 20.8 (**C4**), 23.7 (**C12**), 24.0 (**C2’**), 25.5 (**C8’**), 29.2 (**C9’**), 20.1, 29.5, 29.6, 29.8, 30.2 (5C, 5 × s, **C3’** – **C7’**), 31.4 (**C3**), 37.4 (**C11’**), 39.8 (**C1’**), 70.3 (**C10’**), 74.7 (**C1’’**), 74.9 (**C2**), 117.7 (**C4a**), 123.0, 128.0 (2C, 2 × s, **C7**, **C8**), 126.0 (**C5**), 127.8 (2C, s, **C3’’**), 127.8 (**C5’’**), 128.5 (2C, s, **C4’’**), 138.1 (3C, s, **C25**), 148.0 (**C8a**), 148.2 (**C6**).

#### 10-(3,4-Dihydro-6-hydroxy-2,5,7,8-tetramethyl-chromen-2-yl)decyl methanesulfonate (S7)

To a solution of **S6** (0.221 g, 0.42 mmol) in anhydrous absolute ethanol (50 mL) was added Pd/C (10%). This was stirred under a hydrogen atmosphere for 16 h. The reaction mixture was filtered through a layer of Celite and concentrated *in vacuo*. The crude product was purified by column chromatography on silica gel. Elution with CH_2_Cl_2_ afforded **S7** as a pale yellow solid (0.095 g, 0.21 mmol, 50%). Analysis calcd. for C_24_H_40_O_5_S: C 65.42, H 9.15, S 7.28 found: C 65.62, H 9.34, S 6.99; TLC: R_f_ (CH_2_Cl_2_): 0.11, R_f_ 0.54 (3:1 Et_2_O:CH_2_Cl_2_); m.p. 80.1ºC; LRMS (+ve APCI) *m/z* calcd. for [M]^+^: 441, found: 441; ^1^H NMR (500 MHz, CD_2_Cl_2_) δ (ppm) 1.25 (3H, s, **H12**), 1.29 – 1.39 (10H, m, **H3’** – **H7’**), 1.39 – 1.50 (4H, m, **H2’**, **H8’**), 1.50 – 1.65 (2H, m, **H1’**), 1.74 – 1.88 (4H, m, **H3**, **H9’**), 2.115 (3H, s, **H11**), 2.124 (3H, s, **H9**), 2.17 (3H, s, **H10**), 2.63 (2H, t, *J* = 7.0 Hz, **H4**), 3.02 (3H, s, **H11’**), 4.23 (2H, t, *J* = 6.5 Hz, **H10’**), 4.32 (1H, s (br), **H24**); ^13^C NMR (125 MHz, CD_2_Cl_2_): δ (ppm) 11.4 (**C9**), 11.8 (**C11**), 12.3 (**C10**), 20.1 (**C4**), 23.8(9) (**C12**), 23.9(4) (**C2’**), 25.8 (**C8’**), 29.4 (**C9’**), 29.5, 29.7(7), 29.8(0), 29.9, 30.5 (5C, 5 × s, **C3’** – **C7’**), 32.0 (**C3**), 37.5 (**C11’**), 39.7 (**C1’**), 71.0 (**C10’**), 74.8 (**C2**), 117.8 (**C4a**), 118.2 (**C5**), 121.2, 122.7 (2C, 2 × s, **C7**, **C8**), 145.0 (**C6**), 145.8 (**C8a**).

Scheme for the synthesis of MitoE_11_ precursor **S11:**

#### 1-Bromo-11-(tetrahydro-*2H*-pyran-2-yloxy)-undecane (S8)

A solution of 11-bromoundecanol (2.525 g, 10.05 mmol), DHP (1.4 mL, 1.299 g, 15.44 mmol) and PPTS (0.254 g, 1.01 mmol) in anhydrous CH_2_Cl_2_ was stirred at room temperature for 4 h. The solvents were removed and the residue was dissolved in Et_2_O (50 mL). This was washed with H_2_O (3 × 50 mL), dried over anhydrous MgSO_4_, filtered and concentrated to give a yellow liquid (3.278 g). The crude product was chromatographed on silica gel and **S8** was eluted with 1:9 Et_2_O:hexane as a pale yellow liquid (3.269 g, 9.75 mmol, 97%). ^1^H NMR data were consistent with the literature.^3^

#### 14-(Tetrahydro-*2H*-pyran-2-yloxy)-3-hydroxy-3-methyl-tetradec-1-ene (S9)

To a solution of **S8** (0.337 g, 1.00 mmol) in anhydrous THF (10 mL) stirring at -78ºC was added *t*-BuLi (1.30 M in pentane, 1.50 mL, 2.00 mmol). The reaction was stirred at -78ºC for 1 h. Freshly distilled methyl vinyl ketone (120 μL, 0.139 g, 0.20 mmol) was added to a solution of anhydrous LiBr (0.540 g, 6.29 mmol) in anhydrous THF (5 mL) and left to stir for 5 min and then cooled to -78ºC. The solution of organolithium was added dropwise to the methyl vinyl ketone solution via a dry ice-cooled cannula and the reaction was stirred for 40 min at -78ºC. The reaction was allowed to warm to room temperature over 20 min and then it was poured into saturated aqueous NH_4_Cl (20 mL). Et_2_O (20 mL) was added and then the aqueous phase was extracted with Et_2_O (2 × 20 mL). The combined organic phase was washed with H_2_O (2 × 20 mL), dried over anhydrous MgSO_4_, filtered and concentrated to give a pale yellow oil (0.365 g). The crude product was purified by column chromatography on silica gel and elution with 1:9 Et_2_O:hexane containing 0.1 % Et_3_N afforded **S9** as a colourless oil (0.114 g, 0.35 mmol, 35%). TLC: R_f_ 0.55 (1:19 Et_2_O : petroleum ether 40-60); HRMS (+ve ESI) calcd. for [M+Na]^+^: 349.2713, found 349.2698; ^1^H NMR (300 MHz, CD_2_Cl_2_): δ (ppm) 1.25 (3H, s, **H14**), 1.20 – 1.36 (16H, m, **H5** – **H12**), 1.40 – 1.90 (10H, m, **H4**, **H13**, **H17** – **H19**), 3.37 (1H, dt, *J*_HCH_ = 9.6, *J*_HCCH_ = 6.7 Hz, **H14a**), 3.46 – 3.54 (1H, m, **H20**), 3.72 (1H, dt, *J*_HCH_ = 9.6, *J*_HCCH_ = 6.7 Hz, **H14b**), 3.82 – 3.92 (1H, m, **H20b**), 4.57 (1H, t, *J* = 3.6 Hz, **H15**), 5.03 (ABX system, 1H, dd, *J*_AX_ = 10.8 Hz, *J*_AB_ = 1.5 Hz, **H1a**), 5.19 (ABX system, 1H, dd, *J*_BX_ = 17.4 Hz, *J*_AB_ = 1.5 Hz, **H1b**), 5.91 (ABX system, 1H, dd, *J*_BX_ = 17.4 Hz, *J*_AX_ = 10.8 Hz, **H2**); ^13^C NMR (75 MHz, CD_2_Cl_2_): δ (ppm) 19.7 (**C18**), 25.6 (**C5**), 26.3 (**C19**), 27.7, 29.4, 29.4(6), 29.5(3), 29.0, 29.6, 29.6(5), 29.7(2) (8C, 8 × s , **C6** – **C13**), 30.8 (**C15**), 32.0 (**C17**), 42.5 (**C4**), 62.3 (**C20**), 67.7 (**C14**), 73.3 (**C3**), 98.9 (**C16**), 111.5 (**C1**), 145.3 (**C2**).

#### 2-(11-Hydroxyundecyl)-2,5,7,8-tetramethyl-chromen-6-ol (S10)

A solution of **S9** (0.085 g, 0.26 mmol) and freshly prepared 2,3,5-trimethyl-*p*-hydroquinone (**10**, 0.040 g, 0.27 mmol) in formic acid (2 mL) were heated to reflux and refluxed for 3 h under an atmosphere of nitrogen. The reaction was poured into crushed ice (~ 10 mL) and this was extracted with Et_2_O (3 × 5 mL) under nitrogen. The combined organic phase was washed with H_2_O (3 × 5 mL) under nitrogen, dried over anhydrous MgSO_4_ and concentrated. The residual orange oil was dissolved in MeOH (2 mL), conc. HCl (0.1 mL) was added and the reaction refluxed for a further 30 min under nitrogen. The reaction was diluted with H_2_O (5 mL) and this was extracted with Et_2_O (3 × 5 mL) under nitrogen. The combined organic phase was washed under argon with H_2_O (5 mL), saturated aqueous NaHCO_3_ (2 × 5 mL) and H_2_O again (2 × 5 mL), dried over anhydrous MgSO_4_, filtered and concentrated to give a brown oil which was purified by column chromatography on silica gel and elution with 1:4 Et_2_O:CH_2_Cl_2_ to give **S10** as a pale yellow solid (0.058 g, 0.15 mmol, 58%). Analysis calcd. for C_24_H_40_O_3_: C, 76.55, H, 10.62, found; C, 76.30, H 10.62; TLC: R_f_ 0.64 (1:3 Et_2_O:CH_2_Cl_2_); m.p. 79.2 ºC; HRMS (+ve ESI) calcd. for [M+H]^+^: 377.3050, found 377.3049; HRMS (-ve ESI) calcd. for [M-H]^-^: 375.2905, found 375.2905; ^1^H NMR (500 MHz, CDCl_3_) δ (ppm) 1.22 (3H, s, **H12**), 1.24 – 1.38 (12H, m, **H3’** – **H8’**), 1.32 – 1.38 (2H, m, **H9’**), 1.38 – 1.45 (2H, m, **H2’**), 1.45 – 1.64 (4H, m, **H10’**, **H1’**), 1.72 – 1.85 (2H, m, **H3**), 2.11 (6H, s, **H9**, **H11**), 2.16 (3H, s, **H10**), 2.60 (2H, t, *J* = 7.0 Hz, **H4**), 3.64 (2H, t, *J* = 6.5 Hz, **H11’**), 4.29 (1H, s (br), **H1’’**); ^13^C NMR (125 MHz, CDCl_3_): δ (ppm) 11.3, 11.8 (2C, 2 × s, **C9**, **C11**), 12.3 (**C10**), 20.8 (**C4**), 23.7 (**C2’**), 23.9 (**C12**), 25.8 (**C9’**), 29.5, 29.6(2), 29.6(3), 29.7, 30.2 (6C, 5 × s (br), **C3’** – **C8’**), 31.6 (**C3**), 32.8 (**C10’**), 39.5 (**C1’**), 63.2 (**C11’**), 74.6 (**C2**), 117.4 (**C4a**), 118.6 (**C5**), 121.2 (**C8**), 122.6 (**C7**), 144.6 (**C6**), 145.6 (**C8a**).

#### 11-(6-Hydroxy-2,5,7,8-tetramethyl-chromen-2-yl)undecyl methanesulfonate (S11)

A solution of **S10** (0.200 g, 0.44 mmol) in anhydrous pyridine (3 mL) was stirred at room temperature for 5 min. To this was added MsCl (0.034 mL, 0.050 g, 0.44 mmol) and the resulting solution was stirred for 1 h. The reaction mixture was poured into CH_2_Cl_2_ (10 mL) and this was washed with H_2_O (3 × 10 mL), 1 M HCl (3 × 10 mL) and saturated aqueous NaHCO_3_ (10 mL), dried over anhydrous MgSO_4_, filtered and concentrated to give an orange oil. The crude product was purified by chromatography on silica gel and elution with 1:1 Et_2_O:petroleum ether 40-60 to afford **S11** as a pale yellow solid (0.097 g, 0.21 mmol, 49%).

HRMS (+ve ESI) calcd. for [M+Na]^+^: 477.2645, found 477.2658; TLC: R_f_ 0.54 (3:1 Et_2_O:CH_2_Cl_2_); ^1^H NMR (300 MHz, CDCl_3_) δ (ppm) 1.22 (3H, s, **H12**), 1.22 – 1.48 (16H, m, **H2’** – **H9’**), 1.50 – 1.65 (2H, m, **H1’**), 1.70 – 85 (4H, m, **H3** and **H10’**), 2.11 (6H, s, **H9**, **H11**), 2.16 (3H, s, **H10**), 2.60 (2H, t, *J* = 6.9 Hz, **H4**), 3.00 (3H, s, **H12’**), 4.18 (1H, s (br), **H1’’**), 4.22 (2H, t, *J* = 6.6 Hz, **H11’**).

**8-Hydroxy-octan-2-one (7)**

A solution of Hg(OTf)_2_.(TMU)_2_ was prepared by mixing HgO (01082 g, 0.500 mmol) and Tf_2_O (860 μL, 0.144 g, 0.51 mmol) in CH_3_CN (10.0 mL), stirred for 10 min until it went colourless, then TMU (120 μL, 0.124 g, 1.07 mmol) was added and the solution was stirred for a further 5 min. To this solution was added H_2_O (1.08 mL, 1.08 g, 67.5 mmol) and CH_2_Cl_2_ (4.0 mL) followed by the dropwise addition of a solution of 7-octyn-1-ol (**6**, 1.263 g, 10.00 mmol) in CH_3_CN (1.20 mL) and CH_2_Cl_2_ (0.5 mL) and the reaction was stirred for 48 hours. It was then poured into saturated aqueous NaCl:NaHCO_3_ 1:1 (50 mL) and this was extracted with CH_2_Cl_2_ (3 × 50 mL). The organic phase was dried over anhydrous MgSO_4_, filtered and concentrated to give **7** as a pale yellow oil (1.373 g, 9.52 mmol, 95%). ^1^H NMR data was consistent with the literature^1^ and the crude product was used without further purification.

**8-(Tetrahydro-2H-pyran-2-yloxy)-octan-2-one (8)**

To a solution of **7** (1.360 g, 9.43 mmol) in anhydrous CH_2_Cl_2_ (50 mL) was added dihydropyran (1.36 mL, 1.26 g, 15.00 mmol) and pyridinium *p*-toluene sulfonate (0.377 g, 1.50 mmol) and the reaction was stirred at room temperature for 4 hours. The solvents were removed and the residue was dissolved in Et_2_O (50 mL). This was washed with saturated aqueous NaCl (3 × 50 mL), dried over anhydrous MgSO_4_, filtered and concentrated to give a pale yellow oil. The product was purified by column chromatography on silica gel. Elution with 2:1 petroleum ether 40-60:Et_2_O and afforded **8** as a colourless oil (1.847 g, 8.09 mmol, 86%). ^1^H NMR data were consistent with the literature.^1^

**4-(Tetrahydro-2H-pyran-2-yloxy)-butan-2-one (13).**

A solution of 4-hydroxy-2-butanone (**12**, 2.111 g, 23.96 mmol), dihydropyran (3.20 mL, 2.950 g, 33.07 mmol) and pyridinium *p*-toluene sulfonate (0.592 g, 2.36 mmol) was stirred in anhydrous CH_2_Cl_2_ (30 mL) at room temperature for 4 h. The reaction mixture was then concentrated by removal of the solvents *in vacuo*. The residue was dissolved in Et_2_O (50 mL). This was washed with saturated aqueous NaCl (40 mL) and H_2_O (10 mL), dried over anhydrous MgSO_4_, filtered and concentrated *in vacuo* to give **13** as a pale yellow liquid (4.069 g, 23.63 mmol, 99%). The ^1^H NMR spectrum of the crude product was consistent with that reported in the literature^4^ and the material was used without further purification.

**2-(2-Hydroxyethyl)-2,5,7,8-tetramethyl-chromen-6-ol (15).**

A solution of **14** (0.657 g, 3.28 mmol) and freshly prepared 2,3,5-trimethyl-*p*-hydroquinone (**10**, 0.418 g, 2.75 mmol) in formic acid (60 mL) was heated to reflux and refluxed for 3.5 h under an atmosphere of argon. The reaction was poured onto crushed ice (~ 180 mL) and this was extracted with Et_2_O (3 × 60 mL) under argon. The combined organic phase was washed with H_2_O (3 × 60 mL) under argon, dried over anhydrous MgSO_4_ and concentrated *in vacuo*. The oily brown residue was dissolved in MeOH (6 mL), conc. HCl (0.060 mL) was added and the reaction was refluxed for a further 30 min under argon. The solvents were removed *in vacuo* and the residue was dissolved in Et_2_O (50 mL). This was washed under argon with H_2_O (3 × 50 mL), saturated aqueous NaHCO_3_ (3 × 50 mL) and H_2_O again (3 × 50 mL), dried over anhydrous MgSO_4_, filtered and concentrated to give a brown oil. The crude product was purified by column chromatography on silica gel and eluted with 1:9 Et_2_O:CH_2_Cl_2_ to afford an orange oil (0.406 g) and crystallised from CH_2_Cl_2_ to give **15** as a pale yellow solid (0.306 g, 1.22 mmol, 45%). ^1^H NMR spectroscopic^5^ and m.p.^4^ data were consistent with the literature.

**6-Acetoxyhex-1-yne (17).**

A solution of 5-hexyn-1-ol (**16**, 1.344 g, 13.70 mmol), acetic anhydride (1.406 g, 13.77 mmol) and pyridine (1.223 g, 15.46 mmol) was stirred at room temperature for 3 hours. The reaction was poured into 1 M HCl (20 mL) and this was extracted with Et_2_O (3 × 20 mL). The combined organic phase was washed with saturated aqueous NH_4_Cl (3 × 20 mL), saturated aqueous NaHCO_3_ (20 mL) and saturated aqueous NaCl (20 mL), dried over anhydrous MgSO_4_, filtered and concentrated *in vacuo* to give **17** as a pale yellow liquid (1.906 g, 1.36 mmol, 99%). ^1^H NMR data were consistent with the literature^6^ and the crude product was used without further purification.

**6-Acetoxyhexan-2-one (18).**

A solution of Hg(OTf)_2_.(TMU)_2_ was prepared by mixing HgO (0.070 g, 0.32 mmol) and Tf_2_O (74 μL, 0.124 g, 0.44 mmol) in CH_3_CN (5.1 mL), stirred for 10 min until it went colourless, then TMU (78 μL, 0.075 mg, 0.65 mmol) was added and the solution was stirred for a further 5 min. To this was added H_2_O (0.690 mL, 0.690 g, 38.33 mmol) and then CH_2_Cl_2_ (2.05 mL). A solution of **17** (0.871 g, 6.22 mmol) in CH_3_CN (0.154 mL) and CH_2_Cl_2_ (0.062 mL) was prepared. This was added to the solution of Hg(OTf)_2_.(TMU)_2_ over 5 min and the reaction was left to stir for 48 h. The reaction was poured into saturated aqueous NaCl:NaHCO_3_ 1:1 (20 mL) and this was extracted with Et_2_O (3 × 20 mL). The combined organic phase was dried over anhydrous MgSO_4_, filtered and concentrated to give **18** as a pale yellow liquid (0.913 g, 5.77 mmol, 93%). ^1^H NMR data was consistent with the literature^6^ and the crude product was used without further purification.

**Supplementary references**

1. Dickschat, J. S.; Helmke, E.; Schulz, S. *Chem. Biodivers.* **2005**, *2*, 318-353.

2. Muller, T.; Grandbarbe, L.; Morga, E.; Heuschling, P.; Luu, B. *Bioorg. Med. Chem. Letts.* **2004**, *14*, 6023-6026.

3. Koch, A.; Jonas, U.; Ritter, H.; Spiess, H. W. *Tetrahedron* **2004**, *60*, 10011- 10018.

4. Odinokov, V. N.; Spivak, A. Y.; Emel'yanova, G. A.; Gamalevich, G. D.; Serebryakov, E. P. *Russ. Chem. Bull*. **2001**, *50*, 2121-2129.

5. Nishizawa, M.; Skwarczynski, M.; Imagawa, H.; Sugihara, T. *Chem. Letts.*

**2002**, 12-13.

6. Coxon, J. M.; Hartshorn, M. P.; Swallow, W. H. *J. Org. Chem.* **1974**, *39*, 1142-1148.
